# Racial differences and mortality risk in patients with heart failure and hyponatremia

**DOI:** 10.1371/journal.pone.0218504

**Published:** 2019-06-19

**Authors:** Jeremy A. Miles, Renato Quispe, Yonatan Mehlman, Kavisha Patel, Claudia Lama Von Buchwald, Jee Young You, Seth Sokol, Robert T. Faillace

**Affiliations:** 1 Department of Medicine, Division of General Medicine, Jacobi Medical Center, Albert Einstein College of Medicine, Bronx, New York, United States of America; 2 Department of Medicine, Division of Cardiology, Montefiore Medical Center, Albert Einstein College of Medicine, Bronx, New York, United States of America; 3 Department of Medicine, Division of Cardiology, Rush University Medical Center, Chicago, Illinois, United States of America; Erasmus Medical Center, NETHERLANDS

## Abstract

**Background:**

Hyponatremia is a well-established poor prognostic marker in patients with heart failure. Whether the mortality risk is comparable among different races of patients with heart failure and hyponatremia is unknown.

**Materials and methods:**

Consecutive patients admitted with acute decompensated heart failure and an admission sodium level<135 mEq/L from 1/1/2001 through 12/31/10 were identified. Patients were divided into four groups based on self-reported race: white, African American, Hispanic and other. African Americans were used as the reference group for statistical analysis. The primary outcome was all-cause mortality.

**Results:**

We included 4,343 patients, from which 1,356 (31%) identified as white, 1,248 (29%) as African American, 780 (18%) as Hispanic and 959 (22%) as other. During a median follow-up of 23 months, a total of 2,384 patients died: 678 were African American, 820 were white, 298 were Hispanic and 588 were other. After adjusting for baseline demographics, comorbidities and medication use, Hispanic patients had a 45% less risk of death as compared to African Americans (HR .55, CI .48-.64, p<0.05). There was no difference in mortality between white and African American patients (HR 1.04, CI .92–1.2, p = 0.79).

**Conclusion:**

Hispanic patients admitted for heart failure and who were hyponatremic on admission had an independent lower risk of mortality compared to other groups. These findings may be due to the disparate activity of the renin-angiotensin-aldosterone system among various racial groups. This observational study is hypothesis generating and suggests that treatment of patients with heart failure and hyponatremia should perhaps be focused more on renin-angiotensin-aldosterone system reduction in certain racial groups, yet less in others.

## Introduction

Hyponatremia, defined as a serum sodium level <135 mEq/L, is a well-established marker of poor prognosis in patients with heart failure (HF) and has been described in approximately 20–25% of those admitted to the hospital with acute decompensated heart failure. [1–4] The pathogenesis of hyponatremia in HF is complex but is closely linked to excessive neurohumoral activation, namely increased sympathetic tone and upregulation of the renin-angiotensin-aldosterone system (RAAS).[5]

Studies that have illustrated an association with adverse outcomes and hyponatremia in HF have largely consisted of homogeneous study populations with respect to race. In studies performed in the United States and Europe, most subjects included have been white and a variable percentage have been African American, ranging from 0–40%. [2–4, 6–10] There are studies that have shown an association between hyponatremia and poor outcomes in HF in all-Asian cohorts. [11–13] In addition, there are very few studies in all-Hispanic populations. [14, 15] However, there are no multi-racial cohort studies on this topic that have consisted of a large percentage of Hispanic patients.

The impact of race on HF outcomes is not fully elucidated although it has been studied previously.[16–18] Some of these studies have highlighted an association between race and different RAAS activity. Consequently, it is possible that the clinical significance of hyponatremia in HF differs among racial groups. Thus, the aim of our study was to analyze whether the impact of hyponatremia in a multi-racial population of patients with HF differs based on race, specifically with respect to clinical outcomes and prognosis.

## Materials and methods

### Patient selection

We retrospectively reviewed consecutive patients who were admitted to Montefiore Medical Center for acute decompensated HF and who had a serum sodium level <135 mEq/L on admission from January 1^st^ 2001 through December 31^st^ 2010. Patients were included regardless of etiology or classification of heart failure (i.e. HF with reduced or preserved ejection fraction). Patients <18 years old, those with no sodium level on admission and those with no available data regarding self-reported race were excluded from the study.

Baseline data was collected from the electronic medical record using the hospital’s electronic patient information database (Clinical Looking Glass, Emerging Health Information Technology: Yonkers, NY). Collected data included demographics, comorbidities, medications, admission serum sodium levels, and most recent ejection fraction on transthoracic echocardiogram (TTE). Self-reported race was obtained from documentation in the electronic health record. Patients were divided into four groups based on race: African American, white, Hispanic and other. The ‘other’ group included all races not included in the previous three groups. Patient mortality and readmission data were also obtained from Clinical Looking Glass, which captures all dates of death from the National Death Index and from the hospital’s inpatient record. Readmission data only included readmissions to Montefiore Medical Center. The primary outcome was all-cause mortality. Patients were censored by death and lost-to-follow-up. Survival analysis occurred through December 2011. This study complies with principles declared in the Declaration of Helsinki and was approved by the Albert Einstein College of Medicine Institutional Review Board.

### Statistical analysis

Continuous variables are presented as medians and categorical data is shown as numbers and percentages. Medians were compared using the Kruskal-Wallis test and proportions were compared using the Chi-squared test. Multivariate Cox proportional hazard models were conducted in the overall study population as well as in each race group adjusting for: race, age, sex, diabetes (DM), hypertension (HTN), hyperlipidemia (HLD), chronic kidney disease (CKD), atrial fibrillation (AF), coronary artery disease (CAD), peripheral vascular disease, prior stroke, prior myocardial infarction, chronic obstructive pulmonary disease, malignancy, angiotensin-converting enzyme inhibitor or angiotensin receptor blocker (ARB), beta blocker, spironolactone, digoxin, loop diuretic use, hydralazine/isosorbide dinitrate (ISDN) use, history of implantable cardioverter-defibrillator implantation, inotrope use during admission, serum creatinine levels on admission and ejection fraction (EF). We performed these analyses adjusting for potential confounders (i.e. DM, HTN, PVD etc.) given their known association with both exposure and outcomes.[19–21] Further subgroup analysis was performed stratifying by heart failure with reduced ejection fraction (HFrEF) versus heart failure with preserved ejection fraction (HFpEF). All models were pre-specified in our analysis and p-value of <0.05 was arbitrarily used as cutoff for significance. Comorbidities and medication use were collected at hospital admission. Proportional Hazard assumption was checked using log-log plot of survival. P-values were arbitrarily considered significant if lower than 0.05. Statistical analyses were performed using STATA 14.0.

## Results

A total of 4,343 patients were included in the study, of which 1248 (29%) patients self-identified as African American, 1,356 (31%) as white, 780 (18%) as Hispanic and 959 (22%) patients were categorized as other. Baseline data is summarized in **Table 1** including demographics, comorbidities, medications and EF among the four different racial groups. As shown in **Table 1**, the median sodium level across all groups was 133 mEq/L, with no significant difference among them (p = 0.27). Whites were oldest and had the highest rates of AF, CAD, peripheral vascular disease and chronic obstructive pulmonary disease compared to other groups (p<0.01). They also had the lowest proportion of ACE inhibitor use among the four groups (p<0.01). African American patients were the youngest and had the highest rates of CKD but the lowest rates of CAD and prior myocardial infarctions.

**Table 1:** Baseline characteristics stratified by racial group.

Variable	African American 1,248 (29%)	White 1,356 (31%)	Hispanic 780 (18%)	Other 959 (22%)	p-value
Male, n (%)	584 (47)	641 (47)	341 (44)	487 (49)	0.15
Sodium^a^, mEq/L	133 (132–134)	133 (132–134)	133 (132–134)	133 (132–134)	0.27
Age, years	65 (53–77)	79 (69–87)	68 (59–77)	68 (58–78)	**<0.01**
DM, n (%)	687 (55)	637 (47)	555 (71)	576 (60)	**<0.01**
HTN, n (%)	928 (74)	959 (70)	619 (80)	672 (70)	**<0.01**
HLD, n (%)	237 (19)	175 (13)	211 (27)	139 (14)	**<0.01**
CKD, n (%)	348 (28)	264 (19)	244 (31)	159 (17)	**<0.01**
AF, n (%)	362 (29)	676 (50)	226 (29)	316 (33)	**<0.01**
CAD, n (%)	678 (54)	940 (69)	528 (68)	615 (64)	**<0.01**
PVD, n (%)	189 (15)	234 (17)	127 (16)	125 (13)	**0.04**
Stroke, n (%)	187 (15)	186 (14)	131 (17)	135 (14)	0.25
MI, n (%)	317 (25)	441 (32)	279 (36)	291 (30)	**<0.01**
COPD, n (%)	304 (24)	442 (32)	180 (23)	256 (27)	**<0.01**
Malignancy, n (%)	152 (12)	168 (12)	67 (9)	82 (9)	**<0.01**
ACEI/ARB, n (%)	950 (76)	892 (66)	633 (81)	703 (73)	**<0.01**
Beta Blocker, n (%)	1032 (83)	1042 (77)	662 (85)	751 (78)	**<0.01**
Spironolactone, n (%)	361 (29)	363 (27)	219 (28)	279 (29)	0.56
Digoxin, n (%)	397 (32)	551 (40)	194 (25)	358 (37)	**<0.01**
Hydralazine/ISDN, n (%)	147 (12)	89 (7)	93 (12)	90 (9)	**<0.01**
Loop diuretics, n (%)	998 (90.9)	1,119 (91.6)	625 (89.9)	764 (91.4)	**0.653**
ICD, n (%)	199 (16)	165 (12)	173 (22)	108 (11)	**<0.01**
Inotropes, n (%)	147 (12)	159 (12)	57 (7)	154 (16)	**<0.01**
Creatinine^a^, mg/dL	1.4 (1–2.7)	1.3 (1–1.9)	1.3 (0.9–2.1)	1.4 (1–2.4)	**<0.01**
EF^a^ (%)	40 (25–60)	45 (30–60)	45 (29.25–60)	40 (25–60)	**<0.01**
30-Day Readmission, n (%)	250 (20)	281 (21)	168 (22)	208 (22)	0.77

AF: atrial fibrillation, ACEI/ARB: angiotensin converting enzyme inhibitor/angiotensin receptor blocker CAD: coronary artery disease, CKD: chronic kidney disease, COPD: chronic obstructive pulmonary disease, DM: diabetes mellitus: EF: ejection fraction, HTN: hypertension, HLD: hyperlipidemia, MI: myocardial infarction, ICD: implantable cardioverter-defibrillator, ISDN: isosorbide dinitrate, PVD: peripheral vascular disease.

^a^ Continuous variable are shown as medians (25^th^– 75^th^)

Moreover, Hispanics had a higher proportion of DM, HTN, HLD, CKD, prior myocardial infarction and stroke as compared to the other groups. In addition, Hispanics also had the highest proportions of ACE inhibitor/ARB, beta blocker and implantable cardioverter-defibrillator use among the groups. There was no statistical difference among proportions of 30-day readmissions, ranging from 20–22% in each group (**Table 1**).

As shown in **Table 2**, 2,384 patients died during a median follow-up of 23 months. Of those who died, 678 were African American, 820 were white, 298 were Hispanic and 588 were other. The event rate among Hispanics was 8.5 deaths per 1000 person-months compared to 15.5 deaths per 1000 person-months in African Americans, 21.7 in whites and 20.9 in the other group. In the unadjusted survival analysis, both whites and those who were categorized as ‘other’ had an increased risk of death as compared to African American patients (HR 1.4, CI 1.2–1.5, p<0.001 and HR 1.3, CI 1.2–1.5, p<0.01 respectively). In contrast, Hispanics had 44% less risk of death as compared to African Americans (HR .56, CI .49-.64, p<0.01).

**Table 2:** Event rates per 1000 person-months stratified by racial group.

Race	Person-Months	Number of Deaths	Event Rate	95% Confidence Interval
African American	43863	678	15.5	14.3–16.7
White	37726	820	21.7	20.3–23.2
Hispanic	35110	298	8.5	7.6–9.5
Other	28147	588	20.9	19.2–22.6
Total	144846	2384	16.5	15.8–17.1

After adjusting for all baseline demographic variables, cardiovascular risk factors and medications shown in **Table 3**, Hispanics still had a significantly less risk of death as compared to African Americans (HR .55, CI .48-.64, p<0.01). However, there was no significant difference in mortality risk between whites and African Americans (HR 1.04, CI .92–1.2, p 0.53). Furthermore, the increased risk of death in the ‘other’ group persisted even after multivariate analysis (HR 1.4, CI 1.3–1.6, p<0.01).

**Table 3:** Multivariate cox-proportional hazard models for all-cause mortality in patients with hyponatremia admitted for heart failure.

Variable	Hazard Ratio	95% Confidence Interval	P-value ^a^
African American	REF^b^	REF	
White	1.04	.92–1.2	0.53
Hispanic	.55	.48-.64	**<0.01**
Other	1.4	1.3–1.6	**<0.01**
Male	.99	.90–1.1	0.91
Age	1.03	1.02–1.03	**<0.01**
DM	1.13	1.02–1.2	**0.02**
HTN	.94	.84–1.1	0.3
HLD	.89	.79–1.02	0.09
CKD	1.3	1.2–1.5	**<0.01**
AF	1.2	1.04–1.3	**<0.01**
CAD	.94	.84–1.1	0.31
PVD	1.3	1.1–1.4	**<0.01**
Stroke	1.1	.98–1.3	0.08
MI	1.1	1.01–1.3	**0.03**
COPD	1.3	1.1–1.4	**<0.01**
Malignancy	1.3	1.1–1.5	**<0.01**
ACEI/ARB	.97	.87–1.1	0.58
Beta Blocker	.93	.81–1.1	0.26
Spironolactone	1.1	.96–1.2	0.17
Digoxin	1.1	.97–1.2	0.15
Loop diuretics	1.0	.88–1.2	0.66
Hydralazine/ISDN	1.05	.89–1.2	0.55
Inotropes	1.1	.89–1.3	0.53
Creatinine	1.1	1.1–1.2	**<0.01**
EF	.996	.993-.999	**0.02**

AF: atrial fibrillation, ACEI/ARB: angiotensin converting enzyme inhibitor/angiotensin receptor blocker CAD: coronary artery disease, CKD: chronic kidney disease, COPD: chronic obstructive pulmonary disease, DM: diabetes mellitus: EF: ejection fraction, HTN: hypertension, HLD: hyperlipidemia, MI: myocardial infarction, ICD: implantable cardioverter-defibrillator, ISDN: isosorbide dinitrate, PVD: peripheral vascular disease.

^a^ Bolded values were significant (p<0.05)

^b^ REF: reference, African Americans were used as reference standard

In addition, further multivariate analysis models were performed in patients with hyponatremia stratifying by each group, as shown in **Table 4**. In African American patients, older age (HR 1.02, CI 1.02–1.03, p<0.01) CKD (HR 1.4, CI 1.1–1.7, p<0.01), chronic obstructive pulmonary disease (HR 1.28, CI 1.05–1.55, p = .01), PVD (HR 1.3, CI 1.1–1.7, p = .02) and higher creatinine levels (HR 1.1, CI 1.0–1.1, p<0.01) on admission were significantly associated with higher mortality. Among whites, higher mortality risk was associated with older age (HR 1.03, CI 1.02–1.04, p<0.01), CKD (1.3, CI 1.1–1.7, p = 0.01), malignancy (HR 1.4, CI 1.1–1.8, p<0.01), chronic obstructive pulmonary disease (HR 1.2, CI 1.0–1.4, p = 0.03), prior stroke (HR 1.3, CI 1.0–1.7, p = 0.03), peripheral vascular disease (HR 1.3, CI 1.0–1.6, p = 0.02) and the use of inotropes (HR 1.5, CI 1.1–1.9, p<0.01). Moreover, older age (HR 1.04, CI 1.0–1.1, p<0.01), AF (HR 1.8, CI 1.4–2.4, p<0.01), peripheral vascular disease (HR 1.7, CI 1.2–2.3, p<0.01), hydralazine/ISDN (HR 1.5, CI 1.0–2.1, p = 0.04) and elevated creatinine (HR 1.2, CI 1.1–1.2, p<0.01) were associated with worse mortality among Hispanics.

**Table 4:** Multivariate cox-proportional hazard models for all-cause mortality in patients with hyponatremia admitted for heart failure, stratified by race.

	African American			White			Hispanic			Other		
Variable	HR	95% CI	p-value ^a^	HR	95% CI	p-value ^a^	HR	95% CI	p-value ^a^	HR	95% CI	p-value ^a^
Age	1.02	1.02–1.03	**<0.01**	1.03	1.02–1.04	**<0.01**	1.04	1.0–1.1	**<0.01**	1.02	1.0–1.1	**<0.01**
Sex	1.1	.88–1.25	0.59	1.2	.98–1.4	0.08	.93	.72–1.2	0.61	.80	.66-.98	**0.04**
DM	1.1	.95–1.4	0.14	1.1	.90–1.3	0.44	1.2	.85–1.6	0.37	1.2	.96–1.5	0.10
CKD	1.4	1.1–1.7	**<0.01**	1.3	1.1–1.7	**0.01**	1.3	.91–1.8	0.16	1.4	1.3–1.8	**0.03**
AF	1.1	.89–1.3	0.37	1.1	.93–1.3	0.23	1.8	1.4–2.4	**<0.01**	1.02	.81–1.3	0.89
HTN	.85	.69–1.1	0.14	.88	.72–1.1	0.20	1.1	.75–1.5	0.76	1.2	.94–1.5	0.13
Malignancy	1.3	.97–1.6	0.08	1.4	1.1–1.8	**<0.01**	1.3	.84–1.9	0.24	1.5	1.1–2.1	**0.02**
COPD	1.3	1.05–1.55	**0.01**	1.2	1.0–1.4	**0.03**	1.2	.87–1.6	0.31	1.4	1.1–1.8	**<0.01**
MI	1.1	.9–1.4	0.29	1.03	.84–1.2	.79	1.2	.92–1.7	0.17	1.1	.86–1.4	0.49
Stroke	1.0	.81–1.3	0.73	1.3	1.0–1.7	**0.03**	.96	.71–1.3	0.81	1.01	.77–1.3	0.95
PVD	1.3	1.1–1.7	**0.02**	1.3	1.0–1.6	**0.02**	1.7	1.2–2.3	**<0.01**	1.03	.80–1.3	0.78
CAD	1.0	.85–1.3	0.66	.83	.67–1.0	0.11	1.00	.73–1.4	0.98	.93	.72–1.2	0.56
HLD	.76	.60-.96	**0.02**	.96	.75–1.2	0.74	.83	.62–1.1	0.21	1.01	.78–1.3	0.95
Digoxin	1.1	.90–1.4	0.29	1.1	.87–1.3	0.60	1.02	.75–1.4	0.88	1.2	.96–1.5	0.11
Loop diuretics	1.1	.76–1.4	0.31	1.0	.74–1.4	0.97	1.02	.66–1.6	0.93	.84	.57–1.3	0.39
Spironolactone	.94	.74–1.2	0.61	1.1	.87–1.3	0.55	.99	.72–1.4	0.96	1.4	1.1–1.8	**<0.01**
Hydralazine	1.1	.81–1.4	0.66	.98	.71–1.4	0.91	1.5	1.01–2.1	**0.04**	.89	.65–1.2	0.48
Inotropes	1.5	.75–1.5	0.79	1.5	1.1–1.9	**<0.01**	.74	.40–1.3	0.32	.83	.59–1.2	0.29
BB	.91	.72–1.2	0.45	.95	.76–1.2	0.68	.94	.64–1.4	0.74	.93	.69–1.2	0.63
ACEI/ARB	.86	.69–1.1	0.15	.97	.81–1.2	0.74	.96	.68–1.4	0.82	1.1	.91–1.5	0.27
Creatinine	1.1	1.0–1.1	**<0.01**	1.1	.99–1.1	0.07	1.2	1.1–1.2	**<0.01**	1.1	1.1–1.2	**<0.01**
EF	.99	.99–1.00	0.11	.99	.99–1.00	0.14	.99	.99–1.00	0.39	.99	.99–1.1	0.78

AF: atrial fibrillation, ACEI/ARB: angiotensin converting enzyme inhibitor/angiotensin receptor blocker CAD: coronary artery disease, CKD: chronic kidney disease, COPD: chronic obstructive pulmonary disease, DM: diabetes mellitus: EF: ejection fraction, HTN: hypertension, HLD: hyperlipidemia, MI: myocardial infarction, ICD: implantable cardioverter-defibrillator. PVD: peripheral vascular disease.

^a^ Bolded values were significant (p<0.05)

A subgroup analysis stratifying patients with heart failure with a reduced ejection fraction (HFrEF) and those with heart failure with a preserved ejection fraction (HFpEF) was also performed (**Table 5**). Patients with an ejection fraction <40% on transthoracic echocardiogram were categorized as HFrEF and those with an ejection fraction >40% were considered to have HFpEF. This model illustrated similar outcomes compared to the total study population. Among HFrEF patients, there was no mortality difference among African Americans and whites (HR .98, CI .80–1.2, p = 0.83), there was an increased mortality risk in the other group compared to African Americans (HR 1.3, CI 1.1–1.6, p = 0.01) and Hispanics still had a lower risk of death compared to African American patients (HR .53, CI .42-.67, p<0.01). Similar outcomes were present in patients with HFpEF (Whites HR 1.04, CI .89–1.2, p = 0.62, Hispanics HR .57, CI .47-.69, p<0.01, and other HR 1.5, CI 1.3–1.8, p<0.01).

**Table 5:** Multivariate cox-proportional hazard models for all-cause mortality in patients with hyponatremia admitted for heart failure, stratified by heart failure category.

	Heart Failure with Reduced Ejection Fraction			Heart Failure with Preserved Ejection Fraction		
Variable	HR	95% CI	P-value^a^	HR	95% CI	P-value^a^
African American	REF^b^	REF		REF	REF	
White	.98	.80–1.2	0.83	1.04	.89–1.2	0.62
Hispanic	.53	.42-.67	**<0.01**	.57	.47-.69	**<0.01**
Other	1.3	1.1–1.6	**0.01**	1.5	1.3–1.8	**<0.01**
Male	1.1	.91–1.3	0.40	.98	.86–1.1	0.74
Age	1.03	1.02–1.04	**<0.01**	1.02	1.02–1.03	**<0.01**
DM	1.1	.97–1.3	0.11	1.1	.96–1.2	0.16
HTN	.91	.76–1.1	0.27	.96	.82–1.1	0.64
HLD	.89	.72–1.1	0.28	.92	.79–1.1	0.29
CKD	1.4	1.1–1.7	**<0.01**	1.3	1.1–1.5	<**0.01**
AF	1.2	1.02–1.4	**0.03**	1.1	.97–1.3	0.14
CAD	1.1	.92–1.4	0.26	.86	.74-.99	**0.04**
PVD	1.4	1.2–1.7	**<0.01**	1.2	.98–1.3	0.08
Stroke	.91	.73–1.1	0.43	1.3	1.1–1.4	**<0.01**
MI	1.1	.91–1.3	0.38	1.2	1.01–1.4	**0.04**
COPD	1.2	1.02–1.4	**0.03**	1.2	1.1–1.4	**<0.01**
Malignancy	1.3	.99–1.6	0.06	1.3	1.1–1.6	**<0.01**
ACEI/ARB	1.03	.84–1.3	0.29	.95	.83–1.1	0.44
Beta Blocker	.98	.76–1.3	0.86	.94	.80–1.1	0.43
Spironolactone	.98	.83–1.2	0.83	1.3	1.1–1.5	**<0.01**
Digoxin	1.1	.91–1.3	0.41	1.2	.99–1.4	0.06
Loop diuretics	.93	.71–1.2	0.57	1.1	.89–1.4	0.31
Hydralazine/ISDN	1.03	.83–1.3	0.78	1.1	.80–1.3	0.62
Inotropes	1.05	.85–1.3	0.66	1.1	.80–1.5	0.62
Creatinine	1.1	1.08–1.2	**<0.01**	1.1	1.05–1.13	**<0.01**

AF: atrial fibrillation, ACEI/ARB: angiotensin converting enzyme inhibitor/angiotensin receptor blocker CAD: coronary artery disease, CKD: chronic kidney disease, COPD: chronic obstructive pulmonary disease, DM: diabetes mellitus: EF: ejection fraction, HTN: hypertension, HLD: hyperlipidemia, MI: myocardial infarction, ICD: implantable cardioverter-defibrillator, ISDN: isosorbide dinitrate, PVD: peripheral vascular disease. HR: Hazard Ratio, CI: Confidence Interval

^a^Bolded values were significant (p<0.05)

^b^ REF: reference, African Americans were used as reference standard

## Discussion

The results of our study highlight that patients admitted for acute decompensated HF and who are hyponatremic on admission consist of a high-risk population, yet within this high-risk cohort, the risk of mortality differs based on race. In particular, our data suggests that Hispanics patients with HF and hyponatremia have a lower mortality risk as compared to African Americans, whereas African Americans and whites have similar mortality risk after multivariate adjustment.

The poor prognostic role of hyponatremia in HF has been established in past studies. These studies have illustrated that hyponatremia is associated with multiple adverse outcomes including: worse short and long-term mortality, readmission rates and length of hospital stay.[2–4, 7, 9] However, these trials consisted of rather homogenous study populations and very few analyzed whether race was independent factor associated with poor outcomes. In the United Kingdom Heart Failure Evaluation and Assessment of Risk Trial (UK-HEART), which consisted of chronic ambulatory HF patients, 100% of the cohort was white.[10] In an analysis of hyponatremic patients in the Outcomes of a Prospective Trial of Intravenous Milrinone for Exacerbations of Chronic Heart Failure (OPTIME-CHF) 72% of the cohort was white and 25% were black and race was not a significant predictor of outcomes. [3] Similarly, in the Organized Program to Initiate Lifesaving Treatment in Hospitalized Patients with Heart Failure (OPTIMIZE-HF) registry, approximately 81% of the hyponatremic patients were white and 11% were black.[1] In many of other studies on this topic, race is not even mentioned as a variable in the baseline data. [4, 8, 22–24] What is salient is that Hispanics are poorly represented in mostly all these studies. Our study is unique in that approximately 18% of the patients included were Hispanic, over 1/3^rd^ of whom died during the study period.

The role of race in HF has also been studied previously.[18, 25] However, to our knowledge, the association between race and outcomes among hyponatremic patients with acute HF has not been analyzed before in a large multi-racial cohort. Our study results, which show a reduced mortality risk among Hispanics patients with acute HF and hyponatremia compared to African Americans, are consistent with overall HF mortality data in the United States. The National Center for Health Statistics (NCHS) data on HF mortality from 2000–2014 illustrated a higher age-adjusted HF mortality among African Americans (91.5 deaths per 100,000) as compared to Hispanics (53.3 deaths per 100,000). In addition, whites had a lower age adjusted risk than African Americans but higher than Hispanics (87.3 deaths per 100,000).[26] It is unknown what percentage of these patients included in this data were hyponatremic. Moreover, our analysis included models that were fully-adjusted by several risk factors associated with mortality in HF patients.

Other studies have shown conflicting data with regards to HF outcomes and race, yet, the majority of these studies were different with respect to inclusion criteria and studied outcomes.[18, 25, 27] Our study demonstrates that even within this cohort of severe HF patients, signified by the presence of hyponatremia, race plays a major prognostic role. Our study also provides further data emphasizing the importance in factoring race in management and prognostic decisions and the need for future studies analyzing the impact of race on HF outcomes.

The reduced mortality risk of Hispanics which was found in our analysis needs further study, however there are various explanations which can be suggested. First, our results reflect the idea that a) hyponatremia is closely linked to excessive activation of RAAS and b) RAAS activity has been shown to differ depending on one’s race.[7, 16] For example, African Americans have lower baseline renin levels compared to whites and Hispanics have been shown to have higher plasma renin activity.[28–30] As such, it is possible that hyponatremia in African Americans signifies a more severe state of heart failure as compared to Hispanics given the approach that African Americans ordinarily have less RAAS activation. In contrast, hyponatremia in Hispanics, although implicating a poor prognosis in HF, connotes a less critical state from their higher plasma renin activity.

Moreover, as shown in Table 5, our findings were similar in both patients with HFrEF and HFpEF. Prior studies have illustrated that hyponatremia is a poor prognostic marker in patients with HFpEF as well but they did not analyze the role of race in these findings.[22, 31] While the pathophysiology of HFpEF differs compared to HFrEF, there is still evidence that increased RAAS activation plays a major role.[32, 33] As such, this could explain why, despite the disparate pathophysiology of HFpEF compared to HFrEF, Hispanics with HFpEF have a lower mortality risk compared to other races with HFpEF.

Second, in our study, Hispanics had the highest rates of ACE inhibitor and beta blocker use. Given the circumstance that Hispanics have higher baseline renin levels, it is possible that these medications, which block RAAS activation and the sympathetic nervous system, are more effective in Hispanics as compared to other racial groups and consequently portend an improved survival benefit.[34] While past studies have illustrated a differential survival benefit among African Americans and whites with ACE inhibitors, Hispanics were not included in the study population.[35, 36] That being said, in our study, the use of ACEI and BB did not illustrate a differential survival benefit among the racial groups after multivariate analysis.

A third hypothesis to explain our results relates to the phenomenon known as the ‘Hispanic paradox.’ In our study, despite having higher rates of comorbid cardiovascular risk factors such as HLD, HTN, DM, CKD and prior myocardial infarction, Hispanics still had reduced mortality risk. Multiple epidemiological studies have illustrated that although Hispanics often have higher rates of cardiovascular risk factors and lower socioeconomic status, they have lower cardiovascular and all-cause mortality.[37–39] While this paradox has not been well elucidated and is controversial, our data is consistent with these prior studies which show a lower mortality risk among Hispanics despite a higher risk profile. It is possible that there is some protective genetic or environmental factor that could explain these findings, one that is applicable even in our study cohort of high-risk HF patients with hyponatremia.

### Limitations

There are several important limitations of our study worth noting. 1) The study was retrospective in nature and although many confounding variables were adjusted for in our multivariate analysis, additional confounding factors (i.e. lifestyle or behavioral risk factors, other comorbidities) cannot be excluded. 2) More than 20% of the study population were categorized as other, which consisted of patients of all other races not included in the other three groups of our study. The percentage of Asians or Middle Easterners is unknown in our study. It is important to note that our data showed that the mortality risk of this group was considerably higher compared to African Americans. Yet, this data is difficult to interpret with respect to the racial impact on mortality in this study population given the heterogeneity of this group 3) Being Hispanic is technically not a race, but an ethnicity, and Hispanics can theoretically identify with any racial group. However, in the 2010 census data, almost 40% of Hispanics chose ‘Other Race’ and wrote ‘Latino or Hispanic’ as opposed to choosing one of the races provided such as white or black.[40] Hispanics in our study cohort were only identified as Hispanics yet it is difficult to exclude entirely whether any of these patients were in fact associated with another racial group. 4) A majority of patients did not have brain natriuretic peptide levels measured at admission, which could have been used as a marker for heart failure severity in our study population 5) Mortality data was captured using the National Death Index. As such, there is a possibility that deaths occurring outside the United States were not included, but otherwise there was no loss to follow-up. In addition, we did not have access to disease-specific mortality data and thus do not know what percentage of deaths were due to a cardiovascular cause 6) 30-day readmission data only included readmissions to Montefiore Medical Center and thus is not necessarily an accurate assessment of the impact of race on readmissions in this study population.

## Conclusion

Our study suggests that there is a differential mortality risk based on race in patients with hyponatremia and HF. Particularly, Hispanics with HF and hyponatremia seem to have a lower mortality risk. This may be due to the disparate activity of the RAAS, which plays a major role in the pathophysiology of hyponatremia in HF, among various racial groups. This observational study is hypothesis generating and suggests that treatment of patients with HF and hyponatremia should perhaps be focused more on RAAS reduction in certain racial groups, yet less in others. However, further studies are needed to examine the racial impact on RAAS activity and its effect on clinical outcomes in patients with HF and hyponatremia.

## Supporting information

S1 File Heart failure & hyponatremia cohort dataset. (PDF)

## References

[1] Gheorghiade M, Abraham WT, Albert NM, Gattis Stough W, Greenberg BH, O'Connor CM, et al Relationship between admission serum sodium concentration and clinical outcomes in patients hospitalized for heart failure: an analysis from the OPTIMIZE-HF registry. Eur Heart J. 2007;28(8):980–8. 10.1093/eurheartj/ehl542 . 17309900

[2] Bavishi C, Ather S, Bambhroliya A, Jneid H, Virani SS, Bozkurt B, et al Prognostic significance of hyponatremia among ambulatory patients with heart failure and preserved and reduced ejection fractions. Am J Cardiol. 2014;113(11):1834–8. 10.1016/j.amjcard.2014.03.017 . 24837261

[3] Klein L, O'Connor CM, Leimberger JD, Gattis-Stough W, Pina IL, Felker GM, et al Lower serum sodium is associated with increased short-term mortality in hospitalized patients with worsening heart failure: results from the Outcomes of a Prospective Trial of Intravenous Milrinone for Exacerbations of Chronic Heart Failure (OPTIME-CHF) study. Circulation. 2005;111(19):2454–60. 10.1161/01.CIR.0000165065.82609.3D . 15867182

[4] Gheorghiade M, Rossi JS, Cotts W, Shin DD, Hellkamp AS, Pina IL, et al Characterization and prognostic value of persistent hyponatremia in patients with severe heart failure in the ESCAPE Trial. Arch Intern Med. 2007;167(18):1998–2005. 10.1001/archinte.167.18.1998 . 17923601

[5] Verbrugge FH, Steels P, Grieten L, Nijst P, Tang WH, Mullens W. Hyponatremia in acute decompensated heart failure: depletion versus dilution. J Am Coll Cardiol. 2015;65(5):480–92. 10.1016/j.jacc.2014.12.010 . 25660927

[6] Hauptman PJ, Burnett J, Gheorghiade M, Grinfeld L, Konstam MA, Kostic D, et al Clinical course of patients with hyponatremia and decompensated systolic heart failure and the effect of vasopressin receptor antagonism with tolvaptan. J Card Fail. 2013;19(6):390–7. 10.1016/j.cardfail.2013.04.001 . 23743487

[7] Bettari L, Fiuzat M, Shaw LK, Wojdyla DM, Metra M, Felker GM, et al Hyponatremia and long-term outcomes in chronic heart failure—an observational study from the Duke Databank for Cardiovascular Diseases. J Card Fail. 2012;18(1):74–81. 10.1016/j.cardfail.2011.09.005 . 22196845

[8] Balling L, Schou M, Videbaek L, Hildebrandt P, Wiggers H, Gustafsson F, et al Prevalence and prognostic significance of hyponatraemia in outpatients with chronic heart failure. Eur J Heart Fail. 2011;13(9):968–73. 10.1093/eurjhf/hfr086 . 21743065

[9] DeWolfe A, Lopez B, Arcement LM, Hebert K. Low serum sodium as a poor prognostic indicator for mortality in congestive heart failure patients. Clin Cardiol. 2010;33(12):E13–7. 10.1002/clc.20560 . 21184540 PMC6653197

[10] Kearney MT, Fox KA, Lee AJ, Prescott RJ, Shah AM, Batin PD, et al Predicting death due to progressive heart failure in patients with mild-to-moderate chronic heart failure. J Am Coll Cardiol. 2002;40(10):1801–8. . 12446064 10.1016/s0735-1097(02)02490-7

[11] Yoo BS, Park JJ, Choi DJ, Kang SM, Hwang JJ, Lin SJ, et al Prognostic value of hyponatremia in heart failure patients: an analysis of the Clinical Characteristics and Outcomes in the Relation with Serum Sodium Level in Asian Patients Hospitalized for Heart Failure (COAST) study. Korean J Intern Med. 2015;30(4):460–70. 10.3904/kjim.2015.30.4.460 26161012 PMC4497333

[12] Sato N, Gheorghiade M, Kajimoto K, Munakata R, Minami Y, Mizuno M, et al Hyponatremia and in-hospital mortality in patients admitted for heart failure (from the ATTEND registry). Am J Cardiol. 2013;111(7):1019–25. 10.1016/j.amjcard.2012.12.019 . 23312128

[13] Lu DY, Cheng HM, Cheng YL, Hsu PF, Huang WM, Guo CY, et al Hyponatremia and Worsening Sodium Levels Are Associated With Long-Term Outcome in Patients Hospitalized for Acute Heart Failure. J Am Heart Assoc. 2016;5(3):e002668 10.1161/JAHA.115.002668 27009619 PMC4943243

[14] Arevalo Lorido JC, Carretero Gomez J, Formiga F, Montero Perez-Barquero M, Trullas Vila JC, Aramburu Bodas O, et al Hyponatremia as predictor of worse outcome in real world patients admitted with acute heart failure. Cardiol J. 2013;20(5):506–12. 10.5603/CJ.2013.0136 . 24469874

[15] Mendez-Bailon M, Barba-Martin R, de Miguel-Yanes JM, Zapatero-Gaviria A, Calvo-Porqueras B, Osuna MF, et al Hyponatremia in hospitalised patients with heart failure in internal medicine: Analysis of the Spanish national minimum basic data set (MBDS) (2005–2011). Eur J Intern Med. 2015;26(8):603–6. 10.1016/j.ejim.2015.06.009 . 26118453

[16] Taylor AL, Ziesche S, Yancy C, Carson P, D'Agostino R Jr., Ferdinand K, et al Combination of isosorbide dinitrate and hydralazine in blacks with heart failure. N Engl J Med. 2004;351(20):2049–57. 10.1056/NEJMoa042934 . 15533851

[17] Carson P, Ziesche S, Johnson G, Cohn JN. Racial differences in response to therapy for heart failure: analysis of the vasodilator-heart failure trials. Vasodilator-Heart Failure Trial Study Group. J Card Fail. 1999;5(3):178–87. . 10496190 10.1016/s1071-9164(99)90001-5

[18] Vivo RP, Krim SR, Liang L, Neely M, Hernandez AF, Eapen ZJ, et al Short- and long-term rehospitalization and mortality for heart failure in 4 racial/ethnic populations. J Am Heart Assoc. 2014;3(5):e001134 10.1161/JAHA.114.001134 25324354 PMC4323790

[19] O'Donnell TFX, Powell C, Deery SE, Darling JD, Hughes K, Giles KA, et al Regional variation in racial disparities among patients with peripheral artery disease. J Vasc Surg. 2018;68(2):519–26. 10.1016/j.jvs.2017.10.090 29459014 PMC6057812

[20] Fayfman M, Haw S. Diabetes in Racial and Ethnic Minorities in the United States: Individualizing Approaches to Diagnosis and Management. Curr Diabetes Rev. 2017;13(3):239–50. 10.2174/1573399812666160926142036 . 27678066

[21] Khattar RS, Swales JD, Senior R, Lahiri A. Racial variation in cardiovascular morbidity and mortality in essential hypertension. Heart. 2000;83(3):267–71. 10.1136/heart.83.3.267 10677402 PMC1729353

[22] Rusinaru D, Buiciuc O, Leborgne L, Slama M, Massy Z, Tribouilloy C. Relation of serum sodium level to long-term outcome after a first hospitalization for heart failure with preserved ejection fraction. Am J Cardiol. 2009;103(3):405–10. 10.1016/j.amjcard.2008.09.091 . 19166698

[23] Senni M, De Maria R, Gregori D, Gonzini L, Gorini M, Cacciatore G, et al Temporal trends in survival and hospitalizations in outpatients with chronic systolic heart failure in 1995 and 1999. J Card Fail. 2005;11(4):270–8. . 15880335 10.1016/j.cardfail.2004.11.003

[24] Lee DS, Austin PC, Rouleau JL, Liu PP, Naimark D, Tu JV. Predicting mortality among patients hospitalized for heart failure: derivation and validation of a clinical model. JAMA. 2003;290(19):2581–7. 10.1001/jama.290.19.2581 . 14625335

[25] Durstenfeld MS, Ogedegbe O, Katz SD, Park H, Blecker S. Racial and Ethnic Differences in Heart Failure Readmissions and Mortality in a Large Municipal Healthcare System. JACC Heart Fail. 2016;4(11):885–93. 10.1016/j.jchf.2016.05.008 27395346 PMC5097004

[26] Ni H, Xu J. Recent Trends in Heart Failure-related Mortality: United States, 2000–2014. NCHS Data Brief. 2015;(231):1–8. . 26727546

[27] Ziaeian B, Heidenreich PA, Xu H, DeVore AD, Matsouaka RA, Hernandez AF, et al Race/Ethnic Differences in Outcomes Among Hospitalized Medicare Patients With Heart Failure and Preserved Ejection Fraction. JACC Heart Fail. 2017;5(7):483–93. 10.1016/j.jchf.2017.02.012 28501527 PMC5524150

[28] Rifkin DE, Khaki AR, Jenny NS, McClelland RL, Budoff M, Watson K, et al Association of renin and aldosterone with ethnicity and blood pressure: the Multi-Ethnic Study of Atherosclerosis. Am J Hypertens. 2014;27(6):801–10. 10.1093/ajh/hpt276 24436325 PMC4017931

[29] Sim JJ, Shi J, Calara F, Rasgon S, Jacobsen S, Kalantar-Zadeh K. Association of plasma renin activity and aldosterone-renin ratio with prevalence of chronic kidney disease: the Kaiser Permanente Southern California cohort. J Hypertens. 2011;29(11):2226–35. 10.1097/HJH.0b013e32834bbc8a . 21941205

[30] He J, Klag MJ, Appel LJ, Charleston J, Whelton PK. The renin-angiotensin system and blood pressure: differences between blacks and whites. Am J Hypertens. 1999;12(6):555–62. 10.1016/s0895-7061(99)00030-8 . 10371364

[31] Park JJ, Cho YJ, Oh IY, Park HA, Lee HY, Kim KH, et al Short and long-term prognostic value of hyponatremia in heart failure with preserved ejection fraction versus reduced ejection fraction: An analysis of the Korean Acute Heart Failure registry. Int J Cardiol. 2017;248:239–45. 10.1016/j.ijcard.2017.08.004 . 28821416

[32] Bishu K, Deswal A, Chen HH, LeWinter MM, Lewis GD, Semigran MJ, et al Biomarkers in acutely decompensated heart failure with preserved or reduced ejection fraction. Am Heart J. 2012;164(5):763–70 e3. 10.1016/j.ahj.2012.08.014 23137508 PMC3875288

[33] Borlaug BA. The pathophysiology of heart failure with preserved ejection fraction. Nat Rev Cardiol. 2014;11(9):507–15. 10.1038/nrcardio.2014.83 . 24958077

[34] Eshtehardi P, Pamerla M, Mojadidi MK, Goodman-Meza D, Hovnanians N, Gupta A, et al Addition of angiotensin-converting enzyme inhibitors to beta-blockers has a distinct effect on hispanics compared with african americans and whites with heart failure and reduced ejection fraction: a propensity score-matching study. J Card Fail. 2015;21(6):448–56. 10.1016/j.cardfail.2015.03.010 . 25805065

[35] Investigators S, Yusuf S, Pitt B, Davis CE, Hood WB, Cohn JN. Effect of enalapril on survival in patients with reduced left ventricular ejection fractions and congestive heart failure. N Engl J Med. 1991;325(5):293–302. 10.1056/NEJM199108013250501 . 2057034

[36] Cohn JN, Archibald DG, Ziesche S, Franciosa JA, Harston WE, Tristani FE, et al Effect of vasodilator therapy on mortality in chronic congestive heart failure. Results of a Veterans Administration Cooperative Study. N Engl J Med. 1986;314(24):1547–52. 10.1056/NEJM198606123142404 . 3520315

[37] Medina-Inojosa J, Jean N, Cortes-Bergoderi M, Lopez-Jimenez F. The Hispanic paradox in cardiovascular disease and total mortality. Prog Cardiovasc Dis. 2014;57(3):286–92. 10.1016/j.pcad.2014.09.001 . 25246267

[38] Palloni A, Morenoff JD. Interpreting the paradoxical in the hispanic paradox: demographic and epidemiologic approaches. Ann N Y Acad Sci. 2001;954:140–74. . 11797855 10.1111/j.1749-6632.2001.tb02751.x

[39] Cortes-Bergoderi M, Goel K, Murad MH, Allison T, Somers VK, Erwin PJ, et al Cardiovascular mortality in Hispanics compared to non-Hispanic whites: a systematic review and meta-analysis of the Hispanic paradox. Eur J Intern Med. 2013;24(8):791–9. 10.1016/j.ejim.2013.09.003 . 24095273

[40] Center PR. Is being Hispanic a matter of race, ethnicity or both? 2015, 6 15 Available from: http://www.pewresearch.org/fact-tank/2015/06/15/is-being-hispanic-a-matter-of-race-ethnicity-or-both/.

