# Supplementary material for: Racial differences and mortality risk in patients with heart failure and hyponatremia
Source: PLoS One. 2019 Jun 19;14(6):e0218504. doi: 10.1371/journal.pone.0218504 (PMC6583993; doi:10.1371/journal.pone.0218504)
Supplement: S1 File — (PDF) [file pone.0218504.s001.pdf]

```
----------------------------------------------------------------------
-------------------------------------
      name:  <unnamed>
       log:  /Users/Renato/Desktop/Einstein/Einstein research/
Hyponatremia and HF/6.feb.log
  log type:  text
 opened on:   6 Feb 2018, 19:30:45

. *Let's first look at the population with hyponatremia*

. gen hypona = .
(11957 missing values generated)

. replace hypona = 1 if na<135
(4343 real changes made)

. replace hypona = 0 if na>=135
(7614 real changes made)

.
. label define sodium 0 "Normal/High sodium" 1"Hyponatremia"

. label values hypona sodium

.
. tab hypona

            hypona |      Freq.     Percent        Cum.
-------------------+-----------------------------------
Normal/High sodium |      7,614       63.68       63.68
      Hyponatremia |      4,343       36.32      100.00
-------------------+-----------------------------------
             Total |     11,957      100.00

. *There is a total of 4,343 individuals with hyponatremia

.
. *Now let's look at the frequencies of races among both sodium
groups*

. tab hypona race, col row

+-------------------+
| Key               |
|-------------------|
|     frequency     |
|  row percentage   |
| column percentage |
+-------------------+
```

```
                   |                  Ethnicity
            hypona | African A  Caucasian   Hispanic      Other |
Total
-------------------+--------------------------------------------
+----------
Normal/High sodium |     2,680      2,222      1,468      1,244 |
7,614
                   |     35.20      29.18      19.28      16.34 |
100.00
                   |     68.23      62.10      65.30      56.47 |
63.68
-------------------+--------------------------------------------
+----------
      Hyponatremia |     1,248      1,356        780        959 |
4,343
                   |     28.74      31.22      17.96      22.08 |
100.00
                   |     31.77      37.90      34.70      43.53 |
36.32
-------------------+--------------------------------------------
+----------
             Total |     3,928      3,578      2,248      2,203 |
11,957
                   |     32.85      29.92      18.80      18.42 |
100.00
                   |    100.00     100.00     100.00     100.00 |
100.00


.
. *and just in hyponatremia group..

. tab race if hypona==1

      Ethnicity  |      Freq.     Percent        Cum.
-----------------+-----------------------------------
African American |      1,248       28.74       28.74
       Caucasian |      1,356       31.22       59.96
        Hispanic |        780       17.96       77.92
           Other |        959       22.08      100.00
-----------------+-----------------------------------
           Total |      4,343      100.00

.
. *Now let's look at other variables in these groups (only those with
hyponatremia)*

. tab acearb race if hypona==1, row col chi2

+-------------------+
```

```
| Key                |
|--------------------|
|     frequency      |
|   row percentage   |
| column percentage  |
+--------------------+

           |                     Ethnicity
   ACE/ARB | African A  Caucasian   Hispanic      Other |     Total
-----------+--------------------------------------------+----------
         0 |       298        464        147        256 |     1,165
           |     25.58      39.83      12.62      21.97 |    100.00
           |     23.88      34.22      18.85      26.69 |     26.82
-----------+--------------------------------------------+----------
         1 |       950        892        633        703 |     3,178
           |     29.89      28.07      19.92      22.12 |    100.00
           |     76.12      65.78      81.15      73.31 |     73.18
-----------+--------------------------------------------+----------
     Total |     1,248      1,356        780        959 |     4,343
           |     28.74      31.22      17.96      22.08 |    100.00
           |    100.00     100.00     100.00     100.00 |    100.00

          Pearson chi2(3) =  68.5869   Pr = 0.000

. tab bb race if hypona==1, row col chi2

+--------------------+
| Key                |
|--------------------|
|     frequency      |
|   row percentage   |
| column percentage  |
+--------------------+

           |                     Ethnicity
        BB | African A  Caucasian   Hispanic      Other |     Total
-----------+--------------------------------------------+----------
         0 |       216        314        118        208 |       856
           |     25.23      36.68      13.79      24.30 |    100.00
           |     17.31      23.16      15.13      21.69 |     19.71
-----------+--------------------------------------------+----------
         1 |     1,032      1,042        662        751 |     3,487
           |     29.60      29.88      18.98      21.54 |    100.00
           |     82.69      76.84      84.87      78.31 |     80.29
-----------+--------------------------------------------+----------
     Total |     1,248      1,356        780        959 |     4,343
           |     28.74      31.22      17.96      22.08 |    100.00
           |    100.00     100.00     100.00     100.00 |    100.00

          Pearson chi2(3) =  27.4496   Pr = 0.000
```

```
. tab spiro race if hypona==1, row col chi2

+-------------------+
| Key               |
|-------------------|
|     frequency     |
|  row percentage   |
| column percentage |
+-------------------+

Spironolac |                  Ethnicity
      tone | African A  Caucasian   Hispanic      Other |     Total
-----------+--------------------------------------------+----------
         0 |       887        993        561        680 |     3,121
           |     28.42      31.82      17.98      21.79 |    100.00
           |     71.07      73.23      71.92      70.91 |     71.86
-----------+--------------------------------------------+----------
         1 |       361        363        219        279 |     1,222
           |     29.54      29.71      17.92      22.83 |    100.00
           |     28.93      26.77      28.08      29.09 |     28.14
-----------+--------------------------------------------+----------
     Total |     1,248      1,356        780        959 |     4,343
           |     28.74      31.22      17.96      22.08 |    100.00
           |    100.00     100.00     100.00     100.00 |    100.00

          Pearson chi2(3) =   2.0725   Pr = 0.557

. tab dig race if hypona==1, row col chi2

+-------------------+
| Key               |
|-------------------|
|     frequency     |
|  row percentage   |
| column percentage |
+-------------------+

           |                  Ethnicity
   Digoxin | African A  Caucasian   Hispanic      Other |     Total
-----------+--------------------------------------------+----------
         0 |       851        805        586        601 |     2,843
           |     29.93      28.32      20.61      21.14 |    100.00
           |     68.19      59.37      75.13      62.67 |     65.46
-----------+--------------------------------------------+----------
         1 |       397        551        194        358 |     1,500
           |     26.47      36.73      12.93      23.87 |    100.00
           |     31.81      40.63      24.87      37.33 |     34.54
-----------+--------------------------------------------+----------
     Total |     1,248      1,356        780        959 |     4,343
```

```
           |     28.74      31.22      17.96      22.08 |    100.00 
           |    100.00     100.00     100.00     100.00 |    100.00 

          Pearson chi2(3) =  61.9363   Pr = 0.000

. tab hydra race if hypona==1, row col chi2

+-------------------+
| Key               |
|-------------------|
|     frequency     |
|  row percentage   |
| column percentage |
+-------------------+

hydral/ISD |                   Ethnicity
         N | African A  Caucasian   Hispanic      Other |     Total
-----------+--------------------------------------------+----------
         0 |     1,101      1,267        687        869 |     3,924 
           |     28.06      32.29      17.51      22.15 |    100.00 
           |     88.22      93.44      88.08      90.62 |     90.35 
-----------+--------------------------------------------+----------
         1 |       147         89         93         90 |       419 
           |     35.08      21.24      22.20      21.48 |    100.00 
           |     11.78       6.56      11.92       9.38 |      9.65 
-----------+--------------------------------------------+----------
     Total |     1,248      1,356        780        959 |     4,343 
           |     28.74      31.22      17.96      22.08 |    100.00 
           |    100.00     100.00     100.00     100.00 |    100.00 

          Pearson chi2(3) =  26.0093   Pr = 0.000

. tab aicd race if hypona==1, row col chi2

+-------------------+
| Key               |
|-------------------|
|     frequency     |
|  row percentage   |
| column percentage |
+-------------------+

           |                   Ethnicity
      AICD | African A  Caucasian   Hispanic      Other |     Total
-----------+--------------------------------------------+----------
         0 |     1,049      1,191        607        851 |     3,698 
           |     28.37      32.21      16.41      23.01 |    100.00 
           |     84.05      87.83      77.82      88.74 |     85.15 
-----------+--------------------------------------------+----------
         1 |       199        165        173        108 |       645 
```

```
           |     30.85      25.58      26.82      16.74 |    100.00
           |     15.95      12.17      22.18      11.26 |     14.85
-----------+--------------------------------------------+----------
     Total |     1,248      1,356        780        959 |     4,343
           |     28.74      31.22      17.96      22.08 |    100.00
           |    100.00     100.00     100.00     100.00 |    100.00

          Pearson chi2(3) =  51.7966   Pr = 0.000

. tab inotropes race if hypona==1, row col chi2

+-------------------+
| Key               |
|-------------------|
|     frequency     |
|  row percentage   |
| column percentage |
+-------------------+

           |                 Ethnicity
 Inotropes | African A  Caucasian   Hispanic      Other |     Total
-----------+--------------------------------------------+----------
         0 |     1,101      1,197        723        805 |     3,826
           |     28.78      31.29      18.90      21.04 |    100.00
           |     88.22      88.27      92.69      83.94 |     88.10
-----------+--------------------------------------------+----------
         1 |       147        159         57        154 |       517
           |     28.43      30.75      11.03      29.79 |    100.00
           |     11.78      11.73       7.31      16.06 |     11.90
-----------+--------------------------------------------+----------
     Total |     1,248      1,356        780        959 |     4,343
           |     28.74      31.22      17.96      22.08 |    100.00
           |    100.00     100.00     100.00     100.00 |    100.00

          Pearson chi2(3) =  31.5553   Pr = 0.000

. tab hld race if hypona==1, row col chi2

+-------------------+
| Key               |
|-------------------|
|     frequency     |
|  row percentage   |
| column percentage |
+-------------------+

           |                 Ethnicity
       HLD | African A  Caucasian   Hispanic      Other |     Total
-----------+--------------------------------------------+----------
         0 |     1,011      1,181        569        820 |     3,581
```

```
           |     28.23      32.98      15.89      22.90 |    100.00 
           |     81.01      87.09      72.95      85.51 |     82.45 
-----------+--------------------------------------------+----------
         1 |       237        175        211        139 |       762 
           |     31.10      22.97      27.69      18.24 |    100.00 
           |     18.99      12.91      27.05      14.49 |     17.55 
-----------+--------------------------------------------+----------
     Total |     1,248      1,356        780        959 |     4,343 
           |     28.74      31.22      17.96      22.08 |    100.00 
           |    100.00     100.00     100.00     100.00 |    100.00 

          Pearson chi2(3) =  76.8694   Pr = 0.000

. tab dm race if hypona==1, row col chi2

+-------------------+
| Key               |
|-------------------|
|     frequency     |
|  row percentage   |
| column percentage |
+-------------------+

           |                 Ethnicity
        DM | African A  Caucasian   Hispanic      Other |     Total
-----------+--------------------------------------------+----------
         0 |       561        719        225        383 |     1,888 
           |     29.71      38.08      11.92      20.29 |    100.00 
           |     44.95      53.02      28.85      39.94 |     43.47 
-----------+--------------------------------------------+----------
         1 |       687        637        555        576 |     2,455 
           |     27.98      25.95      22.61      23.46 |    100.00 
           |     55.05      46.98      71.15      60.06 |     56.53 
-----------+--------------------------------------------+----------
     Total |     1,248      1,356        780        959 |     4,343 
           |     28.74      31.22      17.96      22.08 |    100.00 
           |    100.00     100.00     100.00     100.00 |    100.00 

          Pearson chi2(3) = 124.2295   Pr = 0.000

. tab ckd race if hypona==1, row col chi2

+-------------------+
| Key               |
|-------------------|
|     frequency     |
|  row percentage   |
| column percentage |
+-------------------+
```

```
           |                    Ethnicity
       CKD | African A  Caucasian   Hispanic      Other |     Total
-----------+--------------------------------------------+----------
         0 |       900      1,092        536        800 |     3,328 
           |     27.04      32.81      16.11      24.04 |    100.00 
           |     72.12      80.53      68.72      83.42 |     76.63 
-----------+--------------------------------------------+----------
         1 |       348        264        244        159 |     1,015 
           |     34.29      26.01      24.04      15.67 |    100.00 
           |     27.88      19.47      31.28      16.58 |     23.37 
-----------+--------------------------------------------+----------
     Total |     1,248      1,356        780        959 |     4,343 
           |     28.74      31.22      17.96      22.08 |    100.00 
           |    100.00     100.00     100.00     100.00 |    100.00 

          Pearson chi2(3) =  77.6801   Pr = 0.000

. tab af race if hypona==1, row col chi2

+-------------------+
| Key               |
|-------------------|
|     frequency     |
|  row percentage   |
| column percentage |
+-------------------+

           |                    Ethnicity
        AF | African A  Caucasian   Hispanic      Other |     Total
-----------+--------------------------------------------+----------
         0 |       886        680        554        643 |     2,763 
           |     32.07      24.61      20.05      23.27 |    100.00 
           |     70.99      50.15      71.03      67.05 |     63.62 
-----------+--------------------------------------------+----------
         1 |       362        676        226        316 |     1,580 
           |     22.91      42.78      14.30      20.00 |    100.00 
           |     29.01      49.85      28.97      32.95 |     36.38 
-----------+--------------------------------------------+----------
     Total |     1,248      1,356        780        959 |     4,343 
           |     28.74      31.22      17.96      22.08 |    100.00 
           |    100.00     100.00     100.00     100.00 |    100.00 

          Pearson chi2(3) = 159.0115   Pr = 0.000

. tab htn race if hypona==1, row col chi2

+-------------------+
| Key               |
|-------------------|
|     frequency     |
```

```
|  row percentage   |
| column percentage |
+-------------------+
```

| HTN | Ethnicity: African A | Caucasian | Hispanic | Other | Total |
|---|---|---|---|---|---|
| 0 | 320 | 397 | 161 | 287 | 1,165 |
| | 27.47 | 34.08 | 13.82 | 24.64 | 100.00 |
| | 25.64 | 29.28 | 20.64 | 29.93 | 26.82 |
| 1 | 928 | 959 | 619 | 672 | 3,178 |
| | 29.20 | 30.18 | 19.48 | 21.15 | 100.00 |
| | 74.36 | 70.72 | 79.36 | 70.07 | 73.18 |
| Total | 1,248 | 1,356 | 780 | 959 | 4,343 |
| | 28.74 | 31.22 | 17.96 | 22.08 | 100.00 |
| | 100.00 | 100.00 | 100.00 | 100.00 | 100.00 |

Pearson chi2(3) =  24.9428   Pr = 0.000

. tab malig race if hypona==1, row col chi2

```
+-------------------+
| Key               |
|-------------------|
|     frequency     |
|  row percentage   |
| column percentage |
+-------------------+
```

| Malignancy | Ethnicity: African A | Caucasian | Hispanic | Other | Total |
|---|---|---|---|---|---|
| 0 | 1,096 | 1,188 | 713 | 877 | 3,874 |
| | 28.29 | 30.67 | 18.40 | 22.64 | 100.00 |
| | 87.82 | 87.61 | 91.41 | 91.45 | 89.20 |
| 1 | 152 | 168 | 67 | 82 | 469 |
| | 32.41 | 35.82 | 14.29 | 17.48 | 100.00 |
| | 12.18 | 12.39 | 8.59 | 8.55 | 10.80 |
| Total | 1,248 | 1,356 | 780 | 959 | 4,343 |
| | 28.74 | 31.22 | 17.96 | 22.08 | 100.00 |
| | 100.00 | 100.00 | 100.00 | 100.00 | 100.00 |

Pearson chi2(3) =  15.0146   Pr = 0.002

. tab copd race if hypona==1, row col chi2

```
+-------------------+
| Key               |
|-------------------|
|     frequency     |
|  row percentage   |
| column percentage |
+-------------------+

           |                 Ethnicity
      COPD | African A  Caucasian   Hispanic      Other |     Total
-----------+--------------------------------------------+----------
         0 |       944        914        600        703 |     3,161
           |     29.86      28.91      18.98      22.24 |    100.00
           |     75.64      67.40      76.92      73.31 |     72.78
-----------+--------------------------------------------+----------
         1 |       304        442        180        256 |     1,182
           |     25.72      37.39      15.23      21.66 |    100.00
           |     24.36      32.60      23.08      26.69 |     27.22
-----------+--------------------------------------------+----------
     Total |     1,248      1,356        780        959 |     4,343
           |     28.74      31.22      17.96      22.08 |    100.00
           |    100.00     100.00     100.00     100.00 |    100.00

          Pearson chi2(3) =  31.8327   Pr = 0.000

. tab mi race if hypona==1, row col chi2

+-------------------+
| Key               |
|-------------------|
|     frequency     |
|  row percentage   |
| column percentage |
+-------------------+

           |                 Ethnicity
        MI | African A  Caucasian   Hispanic      Other |     Total
-----------+--------------------------------------------+----------
         0 |       931        915        501        668 |     3,015
           |     30.88      30.35      16.62      22.16 |    100.00
           |     74.60      67.48      64.23      69.66 |     69.42
-----------+--------------------------------------------+----------
         1 |       317        441        279        291 |     1,328
           |     23.87      33.21      21.01      21.91 |    100.00
           |     25.40      32.52      35.77      30.34 |     30.58
-----------+--------------------------------------------+----------
     Total |     1,248      1,356        780        959 |     4,343
           |     28.74      31.22      17.96      22.08 |    100.00
           |    100.00     100.00     100.00     100.00 |    100.00
```

```
          Pearson chi2(3) =  28.1001   Pr = 0.000

. tab stroke race if hypona==1, row col chi2

+-------------------+
| Key               |
|-------------------|
|     frequency     |
|  row percentage   |
| column percentage |
+-------------------+

           |                   Ethnicity
    Stroke | African A  Caucasian   Hispanic      Other |     Total
-----------+--------------------------------------------+----------
         0 |     1,061      1,170        649        824 |     3,704 
           |     28.64      31.59      17.52      22.25 |    100.00 
           |     85.02      86.28      83.21      85.92 |     85.29 
-----------+--------------------------------------------+----------
         1 |       187        186        131        135 |       639 
           |     29.26      29.11      20.50      21.13 |    100.00 
           |     14.98      13.72      16.79      14.08 |     14.71 
-----------+--------------------------------------------+----------
     Total |     1,248      1,356        780        959 |     4,343 
           |     28.74      31.22      17.96      22.08 |    100.00 
           |    100.00     100.00     100.00     100.00 |    100.00 

          Pearson chi2(3) =   4.1485   Pr = 0.246

. tab pvd race if hypona==1, row col chi2

+-------------------+
| Key               |
|-------------------|
|     frequency     |
|  row percentage   |
| column percentage |
+-------------------+

           |                   Ethnicity
       PVD | African A  Caucasian   Hispanic      Other |     Total
-----------+--------------------------------------------+----------
         0 |     1,059      1,122        653        834 |     3,668 
           |     28.87      30.59      17.80      22.74 |    100.00 
           |     84.86      82.74      83.72      86.97 |     84.46 
-----------+--------------------------------------------+----------
         1 |       189        234        127        125 |       675 
           |     28.00      34.67      18.81      18.52 |    100.00 
           |     15.14      17.26      16.28      13.03 |     15.54 
-----------+--------------------------------------------+----------
```

```
     Total |     1,248      1,356        780        959 |     4,343 
           |     28.74      31.22      17.96      22.08 |    100.00 
           |    100.00     100.00     100.00     100.00 |    100.00 

          Pearson chi2(3) =   8.1068   Pr = 0.044

. tab cad race if hypona==1, row col chi2

+-------------------+
| Key               |
|-------------------|
|     frequency     |
|  row percentage   |
| column percentage |
+-------------------+

           |                 Ethnicity
       CAD | African A  Caucasian   Hispanic      Other |     Total
-----------+--------------------------------------------+----------
         0 |       570        416        252        344 |     1,582 
           |     36.03      26.30      15.93      21.74 |    100.00 
           |     45.67      30.68      32.31      35.87 |     36.43 
-----------+--------------------------------------------+----------
         1 |       678        940        528        615 |     2,761 
           |     24.56      34.05      19.12      22.27 |    100.00 
           |     54.33      69.32      67.69      64.13 |     63.57 
-----------+--------------------------------------------+----------
     Total |     1,248      1,356        780        959 |     4,343 
           |     28.74      31.22      17.96      22.08 |    100.00 
           |    100.00     100.00     100.00     100.00 |    100.00 

          Pearson chi2(3) =  71.2655   Pr = 0.000

. tab readm race if hypona==1, row col chi2

+-------------------+
| Key               |
|-------------------|
|     frequency     |
|  row percentage   |
| column percentage |
+-------------------+

    30 day |                 Ethnicity
   readmit | African A  Caucasian   Hispanic      Other |     Total
-----------+--------------------------------------------+----------
         0 |       998      1,075        612        751 |     3,436 
           |     29.05      31.29      17.81      21.86 |    100.00 
           |     79.97      79.28      78.46      78.31 |     79.12 
-----------+--------------------------------------------+----------
```

```
          1 |       250        281        168        208 |       907 
            |     27.56      30.98      18.52      22.93 |    100.00 
            |     20.03      20.72      21.54      21.69 |     20.88 
-----------+--------------------------------------------+----------
      Total |     1,248      1,356        780        959 |     4,343 
            |     28.74      31.22      17.96      22.08 |    100.00 
            |    100.00     100.00     100.00     100.00 |    100.00 

          Pearson chi2(3) =   1.1481   Pr = 0.765

.
. tabstat na cr ef, stats (mean sd) by(race)

Summary statistics: mean, sd
  by categories of: race (Ethnicity )

            race |        na        cr        ef
-----------------+------------------------------
African American |  137.7996   1.93516  43.43798
                 |  4.874157  1.789552  16.63788
-----------------+------------------------------
       Caucasian |   137.393  1.615835  45.78944
                 |  5.725957  1.221067  15.50998
-----------------+------------------------------
        Hispanic |  137.4066   1.68716  43.85786
                 |  4.929425  1.541283  16.28942
-----------------+------------------------------
           Other |  136.6428  1.795767  42.33825
                 |  5.103909  1.505105  16.43967
-----------------+------------------------------
           Total |  137.3909   1.76712  43.99412
                 |  5.209118  1.542157  16.26775
------------------------------------------------


.
. foreach var of varlist na cr ef   {
  2. oneway `var' race, bonferroni tabulate
  3. }

            |          Summary of SODIUM
 Ethnicity  |        Mean   Std. Dev.       Freq.
------------+------------------------------------
  African A |   137.79964   4.8741566        3928
  Caucasian |   137.39296   5.7259572        3578
   Hispanic |   137.40658   4.9294255        2248
      Other |   136.64276    5.103909        2203
------------+------------------------------------
      Total |    137.3909   5.2091185       11957

                        Analysis of Variance
```

| Source | SS | df | MS | F | Prob > F |
|---|---|---|---|---|---|
| Between groups | 1889.8734 | 3 | 629.957799 | 23.35 | 0.0000 |
| Within groups | 322535.171 | 11953 | 26.9836168 | | |
| Total | 324425.045 | 11956 | 27.1349151 | | |

Bartlett's test for equal variances: chi2(3) = 116.2662 Prob>chi2 = 0.000

Comparison of SODIUM by Ethnicity
(Bonferroni)

| Row Mean-Col Mean | African | Caucasia | Hispanic |
|---|---|---|---|
| Caucasia | -.406687<br>0.004 | | |
| Hispanic | -.39306<br>0.025 | .013627<br>1.000 | |
| Other | -1.15688<br>0.000 | -.750197<br>0.000 | -.763824<br>0.000 |

| Ethnicity | Summary of Cr: Mean | Std. Dev. | Freq. |
|---|---|---|---|
| African A | 1.9351601 | 1.7895518 | 3905 |
| Caucasian | 1.6158352 | 1.2210672 | 3568 |
| Hispanic | 1.6871601 | 1.541283 | 2243 |
| Other | 1.795767 | 1.5051052 | 2197 |
| Total | 1.76712 | 1.5421575 | 11913 |

Analysis of Variance

| Source | SS | df | MS | F | Prob > F |
|---|---|---|---|---|---|
| Between groups | 208.072209 | 3 | 69.357403 | 29.37 | 0.0000 |
| Within groups | 28121.6387 | 11909 | 2.361377 | | |
| Total | 28329.7109 | 11912 | 2.37824974 | | |

```
Bartlett's test for equal variances:  chi2(3) = 528.2724  Prob>chi2 =
0.000

                        Comparison of Cr by Ethnicity
                                (Bonferroni)
Row Mean-|
Col Mean |   African    Caucasia   Hispanic
---------+---------------------------------
Caucasia |   -.319325
         |      0.000
         |
Hispanic |      -.248    .071325
         |      0.000      0.510
         |
   Other |   -.139393    .179932    .108607
         |      0.004      0.000      0.111

            |             Summary of EF
 Ethnicity  |        Mean   Std. Dev.       Freq.
------------+------------------------------------
  African A |   43.437983   16.637875        3362
  Caucasian |   45.789444    15.50998        2700
   Hispanic |    43.85786   16.289424        2093
      Other |   42.338253    16.43967        1626
------------+------------------------------------
      Total |   43.994121   16.267754        9781

                        Analysis of Variance
    Source              SS         df      MS            F     Prob >
F
----------------------------------------------------------------------
--
Between groups      14239.6194      3   4746.53981     18.03
0.0000
 Within groups      2573937.79   9777   263.264579
----------------------------------------------------------------------
--
    Total           2588177.41   9780   264.639817

Bartlett's test for equal variances:  chi2(3) =  15.5886  Prob>chi2 =
0.001

                        Comparison of EF by Ethnicity
                                (Bonferroni)
Row Mean-|
Col Mean |   African    Caucasia   Hispanic
---------+---------------------------------
Caucasia |    2.35146
         |      0.000
         |
```

```
Hispanic |    .419876   -1.93158
         |      1.000      0.000
         |
   Other |   -1.09973   -3.45119   -1.51961
         |      0.149      0.000      0.028

.
.
. *SURVIVAL ANALYSIS*

. sum time if hypona==1, detail

                            time
-------------------------------------------------------------
      Percentiles      Smallest
 1%            0              0
 5%            0              0
10%            0              0       Obs                4343
25%            5              0       Sum of Wgt.        4343

50%           23                      Mean            33.3516
                        Largest       Std. Dev.      33.31829
75%           51            131
90%           87            131       Variance       1110.108
95%          105            131       Skewness       1.078153
99%          126            131       Kurtosis       3.285295

. *mean follow-up time for them was 33.35 months, median of 23 months

.
. *Now, looking at survival analysis among those with hyponatremia*

. stset time if hypona==1, failure (death==1)

     failure event:  death == 1
obs. time interval:  (0, time]
 exit on or before:  failure
            if exp:  hypona==1

----------------------------------------------------------------------
--------
    11957  total observations
     7614  ignored at outset because of -if <exp>-
      498  observations end on or before enter()
----------------------------------------------------------------------
--------
     3845  observations remaining, representing
     2384  failures in single-record/single-failure data
   144846  total analysis time at risk and under observation
                                              at risk from t =
```

```
0
                                   earliest observed entry t =
0
                                        last observed exit t =
131

.
. stphplot if hypona==1, by(race)

         failure _d:  death == 1
   analysis time _t:  time

. stcoxkm  if hypona==1, by(race)

         failure _d:  death == 1
   analysis time _t:  time

. *Analyzing HRs for mortality by race (unadjusted)*

. stcox i.race

         failure _d:  death == 1
   analysis time _t:  time

Iteration 0:   log likelihood = -18211.738
Iteration 1:   log likelihood = -18100.444
Iteration 2:   log likelihood = -18097.544
Iteration 3:   log likelihood = -18097.538
Refining estimates:
Iteration 0:   log likelihood = -18097.538

Cox regression -- Breslow method for ties

No. of subjects =         3845                     Number of obs   =
3845
No. of failures =         2384
Time at risk    =       144846
                                                   LR chi2(3)      =
228.40
Log likelihood  =   -18097.538                     Prob > chi2     =
0.0000

----------------------------------------------------------------------
--------
          _t | Haz. Ratio   Std. Err.      z    P>|z|     [95% Conf.
Interval]
-------------
+------------------------------------------------------------
        race |
  Caucasian  |   1.363189   .0707948     5.97   0.000     1.231261
```

```
1.509251
   Hispanic  |   .5624133   .0391092    -8.28   0.000     .
4907548    .6445351
      Other  |   1.337037   .0753591     5.15   0.000     1.197201
1.493205
----------------------------------------------------------------------
--------

. stphtest

      Test of proportional-hazards assumption

      Time:  Time
      ----------------------------------------------------------------
                  |                      chi2       df       Prob>chi2
      ------------+---------------------------------------------------
      global test |                     37.30        3         0.0000
      ----------------------------------------------------------------

. sts graph, by(race) ylabel(0.10(0.2)1)
plotregion(fcolor(white))graphregion(fcolor(white)) title("Kaplan-
> Meier Survival Curves") xtitle("Time (months)") legend (label(1
"African American") label(2 "Caucasian")
> label(3 "Hispanic") label(4 "Others")) risktable

         failure _d:  death == 1
   analysis time _t:  time

. sts test race

         failure _d:  death == 1
   analysis time _t:  time


Log-rank test for equality of survivor functions

                 |   Events         Events
race             |  observed       expected
-----------------+-------------------------
African American |       678         718.67
Caucasian        |       820         638.24
Hispanic         |       298         560.68
Other            |       588         466.40
-----------------+-------------------------
Total            |      2384        2384.00

                       chi2(3) =     213.69
                       Pr>chi2 =     0.0000

.
```

```
. *Constructing adjusted models - keep in mind that the population for
all these analysis are individuals w
> ith hyponatremia, and the outcome is mortality*

. *Model 1: Race only

. stcox i.race

         failure _d:  death == 1
   analysis time _t:  time

Iteration 0:   log likelihood = -18211.738
Iteration 1:   log likelihood = -18100.444
Iteration 2:   log likelihood = -18097.544
Iteration 3:   log likelihood = -18097.538
Refining estimates:
Iteration 0:   log likelihood = -18097.538

Cox regression -- Breslow method for ties

No. of subjects =         3845                     Number of obs   =
3845
No. of failures =         2384
Time at risk    =       144846
                                                   LR chi2(3)      =
228.40
Log likelihood  =   -18097.538                     Prob > chi2     =
0.0000

----------------------------------------------------------------------
--------
          _t | Haz. Ratio   Std. Err.      z    P>|z|     [95% Conf.
Interval]
-------------
+----------------------------------------------------------------
        race |
  Caucasian  |   1.363189   .0707948     5.97   0.000     1.231261
1.509251
   Hispanic  |   .5624133   .0391092    -8.28   0.000     .
4907548    .6445351
      Other  |   1.337037   .0753591     5.15   0.000     1.197201
1.493205
----------------------------------------------------------------------
--------

. *Model 2: Race + demographics (age, gender)

. stcox i.race age gender

         failure _d:  death == 1
```

```
   analysis time _t:  time

Iteration 0:   log likelihood = -18211.738
Iteration 1:   log likelihood = -17947.675
Iteration 2:   log likelihood = -17943.209
Iteration 3:   log likelihood = -17943.203
Refining estimates:
Iteration 0:   log likelihood = -17943.203

Cox regression -- Breslow method for ties

No. of subjects =         3845                     Number of obs   =
3845
No. of failures =         2384
Time at risk    =       144846
                                                   LR chi2(5)      =
537.07
Log likelihood  =   -17943.203                     Prob > chi2     =
0.0000

----------------------------------------------------------------------
--------
          _t | Haz. Ratio   Std. Err.      z    P>|z|     [95% Conf.
Interval]
-------------
+-----------------------------------------------------------------
        race |
  Caucasian  |   1.024757   .0553873     0.45   0.651     .9217519
1.139272
   Hispanic  |   .5271491   .0366816    -9.20   0.000     .
4599416    .6041771
      Other  |   1.275561   .0720032     4.31   0.000     1.141964
1.424788
             |
         age |   1.027009   .0016203    16.89   0.000     1.023838
1.030189
      gender |   1.117915   .0475428     2.62   0.009      1.02851
1.215091
----------------------------------------------------------------------
--------

. *Model 3: Model 2 + comorbidities

. stcox i.race age gender dm ckd af htn malig copd mi stroke pvd cad
hld

         failure _d:  death == 1
   analysis time _t:  time

Iteration 0:   log likelihood = -18211.738
```

```
Iteration 1:   log likelihood = -17868.282
Iteration 2:   log likelihood = -17860.747
Iteration 3:   log likelihood = -17860.739
Refining estimates:
Iteration 0:   log likelihood = -17860.739

Cox regression -- Breslow method for ties

No. of subjects =         3845                     Number of obs   =
3845
No. of failures =         2384
Time at risk    =       144846
                                                   LR chi2(16)     =
702.00
Log likelihood  =   -17860.739                     Prob > chi2     =
0.0000

----------------------------------------------------------------------
--------
          _t | Haz. Ratio   Std. Err.      z    P>|z|     [95% Conf.
Interval]
-------------
+----------------------------------------------------------------
        race |
  Caucasian  |   1.025066   .0568041     0.45   0.655     .9195653
1.142671
   Hispanic  |   .5120884   .0358835    -9.55   0.000     .
4463739    .5874773
      Other  |   1.317236   .0754904     4.81   0.000     1.177284
1.473824
             |
         age |   1.025641   .0017023    15.25   0.000      1.02231
1.028983
      gender |   1.066913    .046396     1.49   0.136     .9797462
1.161836
          dm |   1.182184   .0525489     3.77   0.000     1.083549
1.289798
         ckd |   1.416121   .0751301     6.56   0.000     1.276266
1.571301
          af |   1.167737   .0512572     3.53   0.000     1.071475
1.272648
         htn |   .8667471   .0434987    -2.85   0.004       .
78555     .956337
       malig |   1.339149   .0872494     4.48   0.000     1.178612
1.521553
        copd |   1.227417   .0557692     4.51   0.000     1.122837
1.341738
          mi |   1.161625   .0590685     2.95   0.003     1.051435
1.283363
      stroke |   1.127813   .0658656     2.06   0.039     1.005833
```

```
1.264586
         pvd |   1.286374   .0713738     4.54   0.000     1.153822
1.434153
         cad |   .9441892   .0491381    -1.10   0.270     .8526293
1.045581
         hld |   .8958064   .0528771    -1.86   0.062     .7979395
1.005677
----------------------------------------------------------------------
--------

. *Model 4: Model 3 + medications

. stcox i.race age gender dm ckd af htn malig copd mi stroke pvd cad
hld dig spiro hydra inotropes bb acear
> b

         failure _d:  death == 1
   analysis time _t:  time

Iteration 0:   log likelihood = -18211.738
Iteration 1:   log likelihood = -17861.832
Iteration 2:   log likelihood = -17854.122
Iteration 3:   log likelihood = -17854.114
Refining estimates:
Iteration 0:   log likelihood = -17854.114

Cox regression -- Breslow method for ties

No. of subjects =         3845                     Number of obs   =
3845
No. of failures =         2384
Time at risk    =       144846
                                                   LR chi2(22)     =
715.25
Log likelihood  =   -17854.114                     Prob > chi2     =
0.0000

----------------------------------------------------------------------
--------
          _t | Haz. Ratio   Std. Err.      z    P>|z|     [95% Conf.
Interval]
-------------
+-------------------------------------------------------------
        race |
  Caucasian  |   1.014627   .0564789     0.26   0.794     .9097549
1.131587
   Hispanic  |   .5148732   .0361155    -9.46   0.000     .
4487384    .5907549
      Other  |   1.304056   .0749269     4.62   0.000      1.16517
1.459499
```

```
             |
         age |   1.026473   .0017524    15.30   0.000     1.023044
1.029913
      gender |   1.057153   .0461874     1.27   0.203     .9703947
1.151667
          dm |   1.187584    .052931     3.86   0.000     1.088243
1.295993
         ckd |   1.408792   .0770404     6.27   0.000     1.265606
1.568177
          af |   1.128128   .0522279     2.60   0.009      1.03027
1.23528
         htn |   .8816309   .0446294    -2.49   0.013     .
7983583    .9735893
       malig |   1.341957   .0878202     4.49   0.000     1.180414
1.525608
        copd |   1.230085   .0565638     4.50   0.000     1.124071
1.346098
          mi |   1.161683   .0594611     2.93   0.003     1.050797
1.284271
      stroke |   1.139869   .0667286     2.24   0.025     1.016307
1.278453
         pvd |   1.287226   .0716022     4.54   0.000     1.154268
1.435499
         cad |   .9458739    .049507    -1.06   0.288     .8536531
1.048057
         hld |   .8982898   .0531036    -1.81   0.070     .8000121
1.008641
         dig |   1.089498   .0531665     1.76   0.079     .9901222
1.198849
       spiro |    1.03601   .0531037     0.69   0.490     .9369862
1.145499
       hydra |   1.128422   .0828657     1.65   0.100     .9771556
1.303106
   inotropes |   1.099548   .0842054     1.24   0.215     .9462976
1.277617
          bb |   .9928205   .0555787    -0.13   0.898     .8896515
1.107954
      acearb |   .9253182   .0448198    -1.60   0.109     .8415138
1.017468
----------------------------------------------------------------------
--------

. *Model 5: Model 4 + sodium

. stcox i.race age gender dm ckd af htn malig copd mi stroke pvd cad
hld dig spiro hydra inotropes bb acear
> b na

         failure _d:  death == 1
   analysis time _t:  time
```

```
Iteration 0:   log likelihood = -18211.738
Iteration 1:   log likelihood =   -17861.4
Iteration 2:   log likelihood = -17853.675
Iteration 3:   log likelihood = -17853.667
Refining estimates:
Iteration 0:   log likelihood = -17853.667

Cox regression -- Breslow method for ties

No. of subjects =         3845                     Number of obs   =
3845
No. of failures =         2384
Time at risk    =       144846
                                                   LR chi2(23)     =
716.14
Log likelihood  =   -17853.667                     Prob > chi2     =
0.0000

----------------------------------------------------------------------
--------
          _t | Haz. Ratio   Std. Err.      z    P>|z|     [95% Conf.
Interval]
-------------
+---------------------------------------------------------------
        race |
  Caucasian  |    1.01418   .0564577     0.25   0.800     .9093475
1.131097
   Hispanic  |   .5152874   .0361488    -9.45   0.000     .
4490922    .5912397
      Other  |   1.303492   .0748918     4.61   0.000      1.16467
1.458861
             |
         age |   1.026424   .0017527    15.27   0.000     1.022995
1.029865
      gender |   1.057356   .0462025     1.28   0.202     .9705703
1.151902
          dm |   1.187398   .0529186     3.85   0.000      1.08808
1.295781
         ckd |   1.409095   .0770442     6.27   0.000     1.265901
1.568487
          af |   1.128323   .0522292     2.61   0.009     1.030462
1.235478
         htn |   .8819623   .0446511    -2.48   0.013     .
7986495     .973966
       malig |   1.343203   .0879125     4.51   0.000     1.181492
1.527048
        copd |   1.230284   .0565746     4.51   0.000      1.12425
1.346319
          mi |   1.160992   .0594282     2.92   0.004     1.050167
```

```
1.283512
      stroke |   1.140807   .0667972     2.25   0.024      1.01712
1.279535
         pvd |   1.285868   .0715485     4.52   0.000     1.153012
1.434033
         cad |   .9472196   .0495912    -1.04   0.300     .8548432
1.049578
         hld |    .899089   .0531623    -1.80   0.072     .8007039
1.009563
         dig |   1.088573   .0531425     1.74   0.082     .9892431
1.197876
       spiro |   1.036931   .0531769     0.71   0.479     .9377729
1.146574
       hydra |   1.129621   .0829618     1.66   0.097     .9781794
1.304508
   inotropes |   1.096682   .0840711     1.20   0.229      .943687
1.274481
          bb |   .9929726   .0555786    -0.13   0.900      .889803
1.108104
      acearb |    .925303   .0448219    -1.60   0.109     .8414951
1.017458
          na |   .9920761   .0081989    -0.96   0.336      .976136
1.008276
----------------------------------------------------------------------
--------

. *Model 6: Model 5 + creatinine

. stcox i.race age gender dm ckd af htn malig copd mi stroke pvd cad
hld dig spiro hydra inotropes bb acear
> b na cr

         failure _d:  death == 1
   analysis time _t:  time

Iteration 0:   log likelihood = -18176.529
Iteration 1:   log likelihood = -17799.281
Iteration 2:   log likelihood = -17789.848
Iteration 3:   log likelihood = -17789.838
Refining estimates:
Iteration 0:   log likelihood = -17789.838

Cox regression -- Breslow method for ties

No. of subjects =         3838                   Number of obs   =
3838
No. of failures =         2380
Time at risk    =       144369
                                                  LR chi2(24)     =
773.38
```

```
Log likelihood  =   -17789.838                    Prob > chi2     =
0.0000

----------------------------------------------------------------------
--------
          _t | Haz. Ratio   Std. Err.      z    P>|z|     [95% Conf.
Interval]
-------------
+-----------------------------------------------------------------
        race |
  Caucasian  |   1.063107   .0598101     1.09   0.277     .9521128
1.187039
   Hispanic  |   .5417235   .0382425    -8.68   0.000     .
4717238    .6221105
      Other  |    1.35345   .0783096     5.23   0.000     1.208349
1.515975
             |
         age |   1.028975    .001809    16.25   0.000     1.025435
1.032527
      gender |   1.042038   .0456004     0.94   0.347     .9563882
1.135358
          dm |   1.167489   .0520819     3.47   0.001     1.069746
1.274163
         ckd |   1.209623   .0704221     3.27   0.001     1.079181
1.35583
          af |   1.147054   .0532244     2.96   0.003     1.047339
1.256263
         htn |    .916124   .0464112    -1.73   0.084     .8295298
1.011758
       malig |   1.330179   .0872236     4.35   0.000     1.169753
1.512605
        copd |   1.243596   .0573268     4.73   0.000     1.136164
1.361187
          mi |   1.153376   .0591491     2.78   0.005     1.043082
1.275332
      stroke |   1.128771   .0661175     2.07   0.039     1.006345
1.266091
         pvd |   1.250017   .0695596     4.01   0.000     1.120855
1.394064
         cad |   .9369144    .049134    -1.24   0.214     .8453973
1.038339
         hld |   .9145769   .0541044    -1.51   0.131      .814451
1.027012
         dig |   1.118268   .0549615     2.27   0.023     1.015571
1.23135
       spiro |   1.117139   .0585167     2.11   0.034     1.008139
1.237924
       hydra |   1.101984   .0806101     1.33   0.184     .9547946
1.271864
   inotropes |   1.133929    .087032     1.64   0.102     .9755602
```

```
1.318008
          bb |   .9574771   .0537461    -0.77   0.439     .8577245
1.068831
      acearb |   .9504302   .0461294    -1.05   0.295     .8641854
1.045282
          na |   .9918121   .0082938    -0.98   0.326     .9756891
1.008202
          cr |   1.106736   .0137154     8.18   0.000     1.080179
1.133947
----------------------------------------------------------------------
--------

. *Model 7: Model 6 + ejection fraction

. stcox i.race age gender dm ckd af htn malig copd mi stroke pvd cad
hld dig spiro hydra inotropes bb acear
> b na cr ef

         failure _d:  death == 1
   analysis time _t:  time

Iteration 0:   log likelihood = -13936.721
Iteration 1:   log likelihood = -13631.198
Iteration 2:   log likelihood = -13624.817
Iteration 3:   log likelihood = -13624.813
Refining estimates:
Iteration 0:   log likelihood = -13624.813

Cox regression -- Breslow method for ties

No. of subjects =         3137                     Number of obs   =
3137
No. of failures =         1878
Time at risk    =       118740
                                                   LR chi2(25)     =
623.82
Log likelihood  =   -13624.813                     Prob > chi2     =
0.0000

----------------------------------------------------------------------
--------
          _t | Haz. Ratio   Std. Err.      z    P>|z|     [95% Conf.
Interval]
-------------
+---------------------------------------------------------------
        race |
  Caucasian  |   1.040079   .0663013     0.62   0.538     .9179208
1.178494
   Hispanic  |   .5541748   .0413819    -7.90   0.000     .
4787239    .6415175
```

| | | | | | | |
|---|---|---|---|---|---|---|
| Other | 1.42944 | .0936361 | 5.45 | 0.000 | 1.257209 | 1.625265 |
| | | | | | | |
| age | 1.028766 | .0020588 | 14.17 | 0.000 | 1.024739 | 1.032809 |
| gender | .9948354 | .0496494 | -0.10 | 0.917 | .9021322 | 1.097065 |
| dm | 1.130923 | .0569651 | 2.44 | 0.015 | 1.024608 | 1.24827 |
| ckd | 1.322284 | .0834613 | 4.43 | 0.000 | 1.168417 | 1.496414 |
| af | 1.155517 | .0606067 | 2.76 | 0.006 | 1.042632 | 1.280624 |
| htn | .9395921 | .0547658 | -1.07 | 0.285 | .8381574 | 1.053303 |
| malig | 1.29867 | .096283 | 3.52 | 0.000 | 1.123029 | 1.501781 |
| copd | 1.252838 | .0654501 | 4.31 | 0.000 | 1.130907 | 1.387915 |
| mi | 1.129685 | .0646943 | 2.13 | 0.033 | 1.009744 | 1.263874 |
| stroke | 1.118174 | .0727903 | 1.72 | 0.086 | .9842343 | 1.270342 |
| pvd | 1.260316 | .0767195 | 3.80 | 0.000 | 1.118572 | 1.420021 |
| cad | .9401696 | .0566065 | -1.02 | 0.306 | .8355191 | 1.057928 |
| hld | .8997582 | .0573537 | -1.66 | 0.097 | .7940857 | 1.019493 |
| dig | 1.086378 | .0617879 | 1.46 | 0.145 | .9717816 | 1.214488 |
| spiro | 1.083964 | .0646327 | 1.35 | 0.176 | .9644086 | 1.218341 |
| hydra | 1.048051 | .0828586 | 0.59 | 0.553 | .8976077 | 1.223709 |
| inotropes | 1.055645 | .091987 | 0.62 | 0.534 | .889909 | 1.252247 |
| bb | .927722 | .0619705 | -1.12 | 0.261 | .8138771 | 1.057491 |
| acearb | .9693977 | .054496 | -0.55 | 0.580 | .8682615 | 1.082314 |
| na | .9848588 | .0088967 | -1.69 | 0.091 | .967575 | 1.002451 |
| cr | 1.10672 | .0155184 | 7.23 | 0.000 | 1.076719 | 1.137557 |
| ef | .9960676 | .0017002 | -2.31 | 0.021 | .9927407 | .9994055 |

------------------------------------------------------------------------------

```
.
. **Now stratifying by race**

. *African American*

. *Model 2: Race + demographics (age, gender)

. stcox age gender if race==0

         failure _d:  death == 1
   analysis time _t:  time

Iteration 0:   log likelihood = -4377.8632
Iteration 1:   log likelihood = -4335.1856
Iteration 2:   log likelihood = -4335.0325
Iteration 3:   log likelihood = -4335.0325
Refining estimates:
Iteration 0:   log likelihood = -4335.0325

Cox regression -- Breslow method for ties

No. of subjects =         1146                     Number of obs   =
1146
No. of failures =          678
Time at risk    =        43863
                                                   LR chi2(2)      =
85.66
Log likelihood  =   -4335.0325                     Prob > chi2     =
0.0000

----------------------------------------------------------------------
--------
          _t | Haz. Ratio   Std. Err.      z    P>|z|     [95% Conf.
Interval]
-------------
+----------------------------------------------------------------
         age |   1.023497   .0026238     9.06   0.000     1.018368
1.028653
      gender |   1.158383    .091119     1.87   0.062     .9928783
1.351475
----------------------------------------------------------------------
--------

. *Model 3: Model 2 + comorbidities

. stcox age gender dm ckd af htn malig copd mi stroke pvd cad hld if
race==0

         failure _d:  death == 1
   analysis time _t:  time
```

```
Iteration 0:   log likelihood = -4377.8632
Iteration 1:   log likelihood = -4306.0209
Iteration 2:   log likelihood = -4304.9494
Iteration 3:   log likelihood = -4304.9491
Refining estimates:
Iteration 0:   log likelihood = -4304.9491

Cox regression -- Breslow method for ties

No. of subjects =         1146                     Number of obs   =
1146
No. of failures =          678
Time at risk    =        43863
                                                   LR chi2(13)     =
145.83
Log likelihood  =   -4304.9491                     Prob > chi2     =
0.0000

----------------------------------------------------------------------
--------
          _t | Haz. Ratio   Std. Err.      z    P>|z|     [95% Conf.
Interval]
-------------
+----------------------------------------------------------------
         age |    1.02143   .0027726     7.81   0.000      1.01601
1.026878
      gender |   1.099998   .0883611     1.19   0.235     .9397585
1.28756
          dm |   1.146445   .0943433     1.66   0.097     .9756769
1.347101
         ckd |   1.417857   .1288559     3.84   0.000     1.186519
1.6943
          af |   1.093589   .0946461     1.03   0.301     .9229664
1.295754
         htn |   .7644834   .0723787    -2.84   0.005      .
635008    .9203583
       malig |   1.388501   .1632902     2.79   0.005     1.102664
1.748433
        copd |   1.313075   .1157433     3.09   0.002     1.104737
1.560703
          mi |   1.139916   .1174668     1.27   0.204     .9314461
1.395044
      stroke |   1.028273   .1153632     0.25   0.804     .8252988
1.281167
         pvd |   1.329438    .139925     2.71   0.007     1.081628
1.634023
         cad |   1.042719   .0995818     0.44   0.661     .8647208
1.257358
         hld |   .7563647   .0820424    -2.57   0.010     .
```

```
6115077    .9355361
----------------------------------------------------------------------
--------

. *Model 4: Model 3 + medications

. stcox age gender dm ckd af htn malig copd mi stroke pvd cad hld dig
spiro hydra inotropes bb acearb if ra
> ce==0

         failure _d:  death == 1
   analysis time _t:  time

Iteration 0:   log likelihood = -4377.8632
Iteration 1:   log likelihood = -4301.1177
Iteration 2:   log likelihood = -4299.8966
Iteration 3:   log likelihood = -4299.8962
Refining estimates:
Iteration 0:   log likelihood = -4299.8962

Cox regression -- Breslow method for ties

No. of subjects =         1146                     Number of obs   =
1146
No. of failures =          678
Time at risk    =        43863
                                                   LR chi2(19)     =
155.93
Log likelihood  =   -4299.8962                     Prob > chi2     =
0.0000

----------------------------------------------------------------------
--------
         _t | Haz. Ratio   Std. Err.      z    P>|z|     [95% Conf.
Interval]
-------------
+-------------------------------------------------------------
        age |   1.021411   .0028694     7.54   0.000     1.015803
1.027051
     gender |   1.083636   .0875424     0.99   0.320     .9249507
1.269546
         dm |   1.159594   .0959149     1.79   0.073     .9860509
1.363679
        ckd |   1.395662   .1315952     3.54   0.000     1.160169
1.678954
         af |    1.04771   .0942732     0.52   0.604     .8783138
1.249777
        htn |   .7904945   .0751788    -2.47   0.013     .
6560646    .9524696
      malig |    1.37019   .1635492     2.64   0.008     1.084375
```

```
1.73134
        copd |   1.312773   .1175526     3.04   0.002      1.10146
1.564627
          mi |   1.172719   .1218237     1.53   0.125     .9566875
1.437534
      stroke |   1.012622   .1145226     0.11   0.912     .8112986
1.263904
         pvd |   1.362191   .1442306     2.92   0.004     1.106908
1.676348
         cad |   1.043932   .1002837     0.45   0.654     .8647747
1.260206
         hld |    .754333    .082083    -2.59   0.010     .
6094518    .9336561
         dig |   1.191572   .1149141     1.82   0.069     .9863506
1.439492
       spiro |   .9214979   .0944594    -0.80   0.425     .7537733
1.126543
       hydra |   1.112519   .1468204     0.81   0.419     .8589611
1.440926
   inotropes |   1.168332   .1736055     1.05   0.295     .8731403
1.563322
          bb |   .9760134   .1046763    -0.23   0.821     .7909798
1.204332
      acearb |    .827061   .0765329    -2.05   0.040     .
6898756    .9915264
----------------------------------------------------------------------
--------

. *Model 5: Model 4 + sodium

. stcox age gender dm ckd af htn malig copd mi stroke pvd cad hld dig
spiro hydra inotropes bb acearb na if
>  race==0

         failure _d:  death == 1
   analysis time _t:  time

Iteration 0:   log likelihood = -4377.8632
Iteration 1:   log likelihood = -4300.5485
Iteration 2:   log likelihood = -4299.3235
Iteration 3:   log likelihood = -4299.3231
Refining estimates:
Iteration 0:   log likelihood = -4299.3231

Cox regression -- Breslow method for ties

No. of subjects =         1146                     Number of obs   =
1146
No. of failures =          678
Time at risk    =        43863
```

LR chi2(20) = 157.08
Log likelihood = -4299.3231 Prob > chi2 = 0.0000

| _t | Haz. Ratio | Std. Err. | z | P>\|z\| | [95% Conf. | Interval] |
|---|---|---|---|---|---|---|
| age | 1.021373 | .0028709 | 7.52 | 0.000 | 1.015761 | 1.027015 |
| gender | 1.090278 | .0883687 | 1.07 | 0.286 | .9301348 | 1.277992 |
| dm | 1.158619 | .0957984 | 1.78 | 0.075 | .9852816 | 1.36245 |
| ckd | 1.405177 | .1328467 | 3.60 | 0.000 | 1.167502 | 1.691236 |
| af | 1.049902 | .0945428 | 0.54 | 0.589 | .8800323 | 1.25256 |
| htn | .7914121 | .0752764 | -2.46 | 0.014 | .6568093 | .9535996 |
| malig | 1.371571 | .1635433 | 2.65 | 0.008 | 1.085732 | 1.732662 |
| copd | 1.314534 | .1176897 | 3.05 | 0.002 | 1.102971 | 1.566677 |
| mi | 1.169242 | .1215396 | 1.50 | 0.133 | .9537273 | 1.433456 |
| stroke | 1.017635 | .1151501 | 0.15 | 0.877 | .8152196 | 1.270309 |
| pvd | 1.366024 | .1446737 | 2.95 | 0.003 | 1.109963 | 1.681155 |
| cad | 1.043563 | .1002456 | 0.44 | 0.657 | .8644729 | 1.259754 |
| hld | .7555848 | .0822524 | -2.57 | 0.010 | .6104105 | .9352859 |
| dig | 1.188229 | .1146469 | 1.79 | 0.074 | .9834941 | 1.435585 |
| spiro | .9228763 | .0946691 | -0.78 | 0.434 | .7547911 | 1.128392 |
| hydra | 1.114313 | .1471079 | 0.82 | 0.412 | .8602696 | 1.443378 |
| inotropes | 1.163633 | .1731489 | 1.02 | 0.308 | .8692748 | 1.557669 |
| bb | .9743625 | .1044718 | -0.24 | 0.809 | .7896855 | 1.202228 |
| acearb | .8321355 | .0771778 | -1.98 | 0.048 | .6938218 | .998022 |
| na | .9831552 | .0152528 | -1.10 | 0.274 | .9537102 | |

```
1.013509
----------------------------------------------------------------------
--------

. *Model 6: Model 5 + creatinine

. stcox age gender dm ckd af htn malig copd mi stroke pvd cad hld dig
spiro hydra inotropes bb acearb na cr
>  if race==0

         failure _d:  death == 1
   analysis time _t:  time

Iteration 0:   log likelihood = -4351.5195
Iteration 1:   log likelihood = -4268.7299
Iteration 2:   log likelihood =  -4267.164
Iteration 3:   log likelihood = -4267.1636
Refining estimates:
Iteration 0:   log likelihood = -4267.1636

Cox regression -- Breslow method for ties

No. of subjects =         1142                     Number of obs   =
1142
No. of failures =          674
Time at risk    =        43668
                                                   LR chi2(21)     =
168.71
Log likelihood  =   -4267.1636                     Prob > chi2     =
0.0000

----------------------------------------------------------------------
--------
          _t | Haz. Ratio   Std. Err.      z    P>|z|     [95% Conf.
Interval]
-------------
+----------------------------------------------------------------
         age |   1.024006   .0030162     8.05   0.000     1.018112
1.029935
      gender |   1.089092   .0885897     1.05   0.294     .9285932
1.277331
          dm |   1.165707   .0967765     1.85   0.065     .9906558
1.371689
         ckd |   1.231038   .1255163     2.04   0.041     1.008053
1.503349
          af |   1.060895   .0957309     0.66   0.512     .8889213
1.266139
         htn |   .8094279   .0772884    -2.21   0.027     .
6712759    .9760123
       malig |   1.362975   .1627966     2.59   0.010     1.078496
```

```
1.722492
        copd |   1.308478   .1174725     2.99   0.003     1.097356
1.560219
          mi |   1.140733   .1194293     1.26   0.209     .9291101
1.400556
      stroke |   1.018232   .1153498     0.16   0.873     .8154903
1.271377
         pvd |   1.339341   .1419383     2.76   0.006     1.088138
1.648535
         cad |   1.045227   .1007451     0.46   0.646     .8652999
1.262567
         hld |   .7767422   .0847053    -2.32   0.021     .
6272658    .9618386
         dig |   1.222416   .1186112     2.07   0.038     1.010711
1.478465
       spiro |   .9953988    .104773    -0.04   0.965     .8098451
1.223467
       hydra |   1.123341   .1476579     0.88   0.376     .8682108
1.453443
   inotropes |    1.19402   .1781686     1.19   0.235     .8912457
1.599653
          bb |   .9364339   .1010519    -0.61   0.543      .757919
1.156995
      acearb |   .8668424   .0811667    -1.53   0.127     .7215027
1.041459
          na |   .9818774   .0152879    -1.17   0.240     .9523663
1.012303
          cr |   1.069056    .020563     3.47   0.001     1.029503
1.110128
----------------------------------------------------------------------
--------

. *Model 7: Model 6 + ejection fraction

. stcox age gender dm ckd af htn malig copd mi stroke pvd cad hld dig
spiro hydra inotropes bb acearb na cr
>  ef if race==0

         failure _d:  death == 1
   analysis time _t:  time

Iteration 0:   log likelihood = -3539.5051
Iteration 1:   log likelihood = -3472.4346
Iteration 2:   log likelihood = -3471.3737
Iteration 3:   log likelihood = -3471.3735
Refining estimates:
Iteration 0:   log likelihood = -3471.3735

Cox regression -- Breslow method for ties
```

No. of subjects = 973
Number of obs = 973
No. of failures = 563
Time at risk = 37086
LR chi2(22) = 136.26
Log likelihood = -3471.3735
Prob > chi2 = 0.0000

| _t | Haz. Ratio | Std. Err. | z | P>\|z\| | [95% Conf. | Interval] |
|---|---|---|---|---|---|---|
| age | 1.022731 | .0033439 | 6.87 | 0.000 | 1.016198 | 1.029306 |
| gender | 1.048421 | .0941483 | 0.53 | 0.598 | .8792202 | 1.250183 |
| dm | 1.144153 | .1053653 | 1.46 | 0.144 | .9552048 | 1.370476 |
| ckd | 1.366799 | .1508216 | 2.83 | 0.005 | 1.100975 | 1.696804 |
| af | 1.092919 | .108513 | 0.89 | 0.371 | .8996519 | 1.327705 |
| htn | .8543691 | .091747 | -1.47 | 0.143 | .6922113 | 1.054514 |
| malig | 1.253478 | .1659534 | 1.71 | 0.088 | .9669909 | 1.624841 |
| copd | 1.278349 | .1258815 | 2.49 | 0.013 | 1.053974 | 1.55049 |
| mi | 1.128883 | .12967 | 1.06 | 0.291 | .9013118 | 1.413914 |
| stroke | 1.043111 | .1282649 | 0.34 | 0.731 | .8197163 | 1.327387 |
| pvd | 1.319862 | .1539198 | 2.38 | 0.017 | 1.050178 | 1.6588 |
| cad | 1.048136 | .1129318 | 0.44 | 0.663 | .8486028 | 1.294585 |
| hld | .7613017 | .0895099 | -2.32 | 0.020 | .6046123 | .9585983 |
| dig | 1.123664 | .1251201 | 1.05 | 0.295 | .9033483 | 1.397713 |
| spiro | .9420735 | .1098077 | -0.51 | 0.609 | .7496681 | 1.18386 |
| hydra | 1.063398 | .1497063 | 0.44 | 0.662 | .8069804 | 1.401293 |
| inotropes | 1.04593 | .1760612 | 0.27 | 0.790 | .7520035 | 1.454739 |
| bb | .9110815 | .1112538 | -0.76 | 0.446 | .717159 | |

```
1.157441
      acearb |   .8597759   .0905774    -1.43   0.152     .6993767
1.056962
          na |   .9700982   .0161741    -1.82   0.069     .9389099
1.002323
          cr |   1.078522   .0230958     3.53   0.000     1.034192
1.124752
          ef |   .9949636   .0031757    -1.58   0.114     .9887588
1.001207
----------------------------------------------------------------------
--------


.
. *Caucasian*

. *Model 2: Race + demographics (age, gender)

. stcox age gender if race==1

         failure _d:  death == 1
   analysis time _t:  time

Iteration 0:   log likelihood = -5190.4116
Iteration 1:   log likelihood = -5131.1085
Iteration 2:   log likelihood =  -5129.448
Iteration 3:   log likelihood = -5129.4466
Refining estimates:
Iteration 0:   log likelihood = -5129.4466

Cox regression -- Breslow method for ties

No. of subjects =         1131                     Number of obs   =
1131
No. of failures =          820
Time at risk    =        37726
                                                   LR chi2(2)      =
121.93
Log likelihood  =   -5129.4466                     Prob > chi2     =
0.0000

----------------------------------------------------------------------
--------
          _t | Haz. Ratio   Std. Err.      z    P>|z|     [95% Conf.
Interval]
-------------
+----------------------------------------------------------------
         age |   1.033498   .0032719    10.41   0.000     1.027105
1.039931
      gender |   1.349625   .0991559     4.08   0.000     1.168627
1.558656
```

```
------------------------------------------------------------------------------
--------

. *Model 3: Model 2 + comorbidities

. stcox age gender dm ckd af htn malig copd mi stroke pvd cad hld if
race==1

         failure _d:  death == 1
   analysis time _t:  time

Iteration 0:   log likelihood = -5190.4116
Iteration 1:   log likelihood = -5107.9773
Iteration 2:   log likelihood = -5105.2184
Iteration 3:   log likelihood = -5105.2144
Refining estimates:
Iteration 0:   log likelihood = -5105.2144

Cox regression -- Breslow method for ties

No. of subjects =         1131                     Number of obs   =
1131
No. of failures =          820
Time at risk    =        37726
                                                   LR chi2(13)     =
170.39
Log likelihood  =   -5105.2144                     Prob > chi2     =
0.0000

------------------------------------------------------------------------------
--------
          _t | Haz. Ratio   Std. Err.      z    P>|z|     [95% Conf.
Interval]
-------------
+----------------------------------------------------------------
         age |   1.034413   .0034963    10.01   0.000     1.027583
1.041289
      gender |   1.266586   .0962633     3.11   0.002     1.091293
1.470035
          dm |   1.145119   .0860061     1.80   0.071     .9883704
1.326727
         ckd |   1.342526   .1294052     3.06   0.002     1.111414
1.621696
          af |   1.145502   .0830438     1.87   0.061     .9937734
1.320395
         htn |   .8196865   .0673531    -2.42   0.016     .
6977584    .9629206
       malig |   1.286247   .1403062     2.31   0.021     1.038661
1.592851
        copd |   1.168045   .0884396     2.05   0.040     1.006955
```

```
1.354906
          mi |    1.08798   .0918504     1.00   0.318     .9220621
1.283754
      stroke |   1.232652   .1277812     2.02   0.044     1.006009
1.510355
         pvd |   1.350747   .1273875     3.19   0.001     1.122789
1.624988
         cad |   .8412562   .0758524    -1.92   0.055     .7049838
1.00387
         hld |   .9219445    .102908    -0.73   0.467     .7407866
1.147404
----------------------------------------------------------------------
--------

. *Model 4: Model 3 + medications

. stcox age gender dm ckd af htn malig copd mi stroke pvd cad hld dig
spiro hydra inotropes bb acearb if ra
> ce==1

         failure _d:  death == 1
   analysis time _t:  time

Iteration 0:   log likelihood = -5190.4116
Iteration 1:   log likelihood = -5101.3271
Iteration 2:   log likelihood = -5098.4436
Iteration 3:   log likelihood =  -5098.439
Refining estimates:
Iteration 0:   log likelihood =  -5098.439

Cox regression -- Breslow method for ties

No. of subjects =         1131                     Number of obs   =
1131
No. of failures =          820
Time at risk    =        37726
                                                   LR chi2(19)     =
183.95
Log likelihood  =    -5098.439                     Prob > chi2     =
0.0000

----------------------------------------------------------------------
--------
          _t | Haz. Ratio   Std. Err.      z    P>|z|     [95% Conf.
Interval]
-------------
+------------------------------------------------------------------
         age |   1.036913   .0036185    10.39   0.000     1.029845
1.04403
      gender |   1.229933   .0947535     2.69   0.007      1.05756
```

```
1.4304
          dm |   1.137003    .085445     1.71   0.088     .9812832
1.317434
         ckd |   1.334502   .1318391     2.92   0.003      1.09958
1.619615
          af |   1.111267   .0858987     1.36   0.172      .955041
1.293047
         htn |   .8517789   .0714048    -1.91   0.056     .7227207
1.003883
       malig |   1.289964   .1414677     2.32   0.020     1.040466
1.599289
        copd |   1.179477   .0903351     2.16   0.031     1.015072
1.37051
          mi |   1.058453   .0907152     0.66   0.507     .8947855
1.252057
      stroke |   1.288108   .1347256     2.42   0.015     1.049358
1.581179
         pvd |   1.323697   .1257863     2.95   0.003     1.098757
1.594686
         cad |   .8378014   .0768824    -1.93   0.054     .6998888
1.00289
         hld |   .9251364   .1038392    -0.69   0.488     .7424465
1.15278
         dig |   1.054991    .085239     0.66   0.508     .9004826
1.236012
       spiro |   1.085628   .0946039     0.94   0.346      .915178
1.287824
       hydra |   1.106562   .1594872     0.70   0.482     .8342441
1.46777
   inotropes |   1.458798   .1852496     2.97   0.003     1.137373
1.871058
          bb |   1.039418   .0967873     0.42   0.678     .8660224
1.247531
      acearb |   .9585019   .0749762    -0.54   0.588     .8222616
1.117316
----------------------------------------------------------------------
--------

. *Model 5: Model 4 + sodium

. stcox age gender dm ckd af htn malig copd mi stroke pvd cad hld dig
spiro hydra inotropes bb acearb na if
>  race==1

         failure _d:  death == 1
   analysis time _t:  time

Iteration 0:   log likelihood = -5190.4116
Iteration 1:   log likelihood = -5101.3228
Iteration 2:   log likelihood = -5098.4396
```

```
Iteration 3:   log likelihood =  -5098.435
Refining estimates:
Iteration 0:   log likelihood =  -5098.435

Cox regression -- Breslow method for ties

No. of subjects =         1131                     Number of obs   =
1131
No. of failures =          820
Time at risk    =        37726
                                                   LR chi2(20)     =
183.95
Log likelihood  =    -5098.435                     Prob > chi2     =
0.0000

----------------------------------------------------------------------
--------
          _t | Haz. Ratio   Std. Err.      z    P>|z|     [95% Conf.
Interval]
-------------
+-----------------------------------------------------------------
         age |   1.036921   .0036197    10.39   0.000      1.02985
1.044039
      gender |   1.229966   .0947578     2.69   0.007     1.057586
1.430443
          dm |   1.137076    .085453     1.71   0.087      .981342
1.317524
         ckd |   1.334318   .1318395     2.92   0.004     1.099398
1.619435
          af |   1.111204   .0859023     1.36   0.173     .9549729
1.292994
         htn |   .8519231   .0714333    -1.91   0.056     .7228158
1.004091
       malig |   1.290277    .141548     2.32   0.020     1.040647
1.59979
        copd |   1.179453   .0903332     2.16   0.031     1.015051
1.370482
          mi |   1.058665   .0907649     0.66   0.506     .8949122
1.252381
      stroke |   1.288142   .1347293     2.42   0.015     1.049385
1.581221
         pvd |   1.323552   .1257823     2.95   0.003     1.098621
1.594535
         cad |   .8375815   .0769039    -1.93   0.054     .6996368
1.002724
         hld |   .9247999   .1038708    -0.70   0.486     .7420674
1.15253
         dig |   1.055208   .0852899     0.66   0.506     .9006115
1.236342
       spiro |   1.085424   .0946067     0.94   0.347     .9149723
```

```
1.28763
       hydra |   1.106797   .1595341     0.70   0.481     .8344018
1.468116
   inotropes |   1.459571    .185541     2.97   0.003     1.137681
1.872536
          bb |   1.039164   .0968063     0.41   0.680     .8657405
1.247326
      acearb |   .9583892   .0749798    -0.54   0.587     .8221442
1.117213
          na |    1.00123   .0138242     0.09   0.929     .9744983
1.028695
----------------------------------------------------------------------
--------

. *Model 6: Model 5 + creatinine

. stcox age gender dm ckd af htn malig copd mi stroke pvd cad hld dig
spiro hydra inotropes bb acearb na cr
>  if race==1

         failure _d:  death == 1
   analysis time _t:  time

Iteration 0:   log likelihood = -5190.4116
Iteration 1:   log likelihood = -5096.4619
Iteration 2:   log likelihood = -5093.2195
Iteration 3:   log likelihood = -5093.2133
Iteration 4:   log likelihood = -5093.2133
Refining estimates:
Iteration 0:   log likelihood = -5093.2133

Cox regression -- Breslow method for ties

No. of subjects =         1131                     Number of obs   =
1131
No. of failures =          820
Time at risk    =        37726
                                                   LR chi2(21)     =
194.40
Log likelihood  =   -5093.2133                     Prob > chi2     =
0.0000

----------------------------------------------------------------------
--------
          _t | Haz. Ratio   Std. Err.      z    P>|z|     [95% Conf.
Interval]
-------------
+---------------------------------------------------------------
         age |   1.038231   .0036486    10.68   0.000     1.031104
1.045407
```

```
      gender |   1.216721   .0936089     2.55   0.011     1.046414
1.414747
          dm |   1.100901   .0835338     1.27   0.205     .9487707
1.277426
         ckd |   1.221163   .1260906     1.94   0.053     .9974323
1.495079
          af |   1.127158   .0871573     1.55   0.122     .9686476
1.311607
         htn |   .8964845   .0764097    -1.28   0.200     .7585648
1.05948
       malig |    1.24903   .1382302     2.01   0.045     1.005473
1.551584
        copd |   1.178894   .0905266     2.14   0.032     1.014171
1.37037
          mi |   1.082485   .0931489     0.92   0.357     .9144819
1.281352
      stroke |   1.262829   .1325542     2.22   0.026      1.02801
1.551286
         pvd |   1.313727   .1248899     2.87   0.004     1.090399
1.582796
         cad |   .8157015   .0752919    -2.21   0.027     .
6807107     .977462
         hld |    .932209   .1047252    -0.62   0.532     .7479775
1.161818
         dig |   1.063922   .0860211     0.77   0.443     .9080041
1.246614
       spiro |   1.149839   .1026001     1.56   0.118     .9653495
1.369588
       hydra |    1.06158   .1534954     0.41   0.679     .7996065
1.409384
   inotropes |   1.456297   .1849268     2.96   0.003     1.135431
1.867837
          bb |   1.013634   .0947241     0.14   0.885     .8439883
1.21738
      acearb |   .9577998   .0749702    -0.55   0.582     .8215772
1.116609
          na |   1.000211   .0139881     0.02   0.988     .9731668
1.028006
          cr |   1.105913   .0329831     3.38   0.001      1.04312
1.172485
----------------------------------------------------------------------
--------

. *Model 7: Model 6 + ejection fraction

. stcox age gender dm ckd af htn malig copd mi stroke pvd cad hld dig
spiro hydra inotropes bb acearb na cr
>  ef if race==1

         failure _d:  death == 1
```

```
   analysis time _t:  time

Iteration 0:   log likelihood = -3648.5678
Iteration 1:   log likelihood = -3576.0063
Iteration 2:   log likelihood =   -3574.03
Iteration 3:   log likelihood = -3574.0281
Refining estimates:
Iteration 0:   log likelihood = -3574.0281

Cox regression -- Breslow method for ties

No. of subjects =          860                     Number of obs   =
860
No. of failures =          604
Time at risk    =        29222
                                                   LR chi2(22)     =
149.08
Log likelihood  =   -3574.0281                     Prob > chi2     =
0.0000

----------------------------------------------------------------------
--------
          _t | Haz. Ratio   Std. Err.      z    P>|z|     [95% Conf.
Interval]
-------------
+---------------------------------------------------------------
         age |   1.036927   .0041978     8.96   0.000     1.028732
1.045187
      gender |   1.177775   .1104982     1.74   0.081     .9799484
1.415539
          dm |   1.070015    .093374     0.78   0.438     .9018007
1.269607
         ckd |   1.333915   .1523385     2.52   0.012     1.066394
1.668548
          af |   1.115313   .1014172     1.20   0.230     .9332448
1.332901
         htn |     .88059   .0880617    -1.27   0.204     .7238541
1.071264
       malig |   1.398327   .1791138     2.62   0.009      1.08787
1.797382
        copd |   1.212863   .1091734     2.14   0.032     1.016699
1.446875
          mi |   1.026244   .1018121     0.26   0.794     .8448975
1.246513
      stroke |    1.31481   .1611227     2.23   0.026     1.034077
1.671756
         pvd |   1.292368   .1397509     2.37   0.018     1.045541
1.597465
         cad |   .8347662   .0937304    -1.61   0.108     .6698677
1.040257
```

```
         hld |   .9595515   .1188869    -0.33   0.739     .7526719
1.223294
         dig |   1.051807   .1019583     0.52   0.602     .8698091
1.271887
       spiro |   1.064483   .1101409     0.60   0.546     .8690918
1.303802
       hydra |   .9806516   .1623715    -0.12   0.906      .708887
1.356602
   inotropes |   1.494231   .2154024     2.79   0.005      1.12645
1.982091
          bb |   .9527202   .1115618    -0.41   0.679     .7573406
1.198504
      acearb |   .9695292   .0913757    -0.33   0.743     .8060042
1.166231
          na |   .9976938   .0154831    -0.15   0.882     .9678044
1.028506
          cr |   1.065467    .037873     1.78   0.074     .9937643
1.142344
          ef |   .9952926   .0031643    -1.48   0.138     .9891099
1.001514
----------------------------------------------------------------------
--------

.
. *Hispanic*

. *Model 2: Race + demographics (age, gender)

. stcox age gender if race==2

         failure _d:  death == 1
   analysis time _t:  time

Iteration 0:   log likelihood = -1786.4702
Iteration 1:   log likelihood = -1753.3324
Iteration 2:   log likelihood =  -1752.504
Iteration 3:   log likelihood = -1752.5037
Refining estimates:
Iteration 0:   log likelihood = -1752.5037

Cox regression -- Breslow method for ties

No. of subjects =          755                     Number of obs   =
755
No. of failures =          298
Time at risk    =        35110
                                                   LR chi2(2)      =
67.93
Log likelihood  =   -1752.5037                     Prob > chi2     =
0.0000
```

```
------------------------------------------------------------------------------
--------
          _t | Haz. Ratio   Std. Err.      z    P>|z|     [95% Conf.
Interval]
-------------
+----------------------------------------------------------------
         age |   1.039054   .0050888     7.82   0.000     1.029128
1.049076
      gender |   1.040818    .125313     0.33   0.740     .8220367
1.317826
------------------------------------------------------------------------------
--------

. *Model 3: Model 2 + comorbidities

. stcox age gender dm ckd af htn malig copd mi stroke pvd cad hld if
race==2

         failure _d:  death == 1
   analysis time _t:  time

Iteration 0:   log likelihood = -1786.4702
Iteration 1:   log likelihood = -1726.2566
Iteration 2:   log likelihood = -1723.9509
Iteration 3:   log likelihood = -1723.9479
Iteration 4:   log likelihood = -1723.9479
Refining estimates:
Iteration 0:   log likelihood = -1723.9479

Cox regression -- Breslow method for ties

No. of subjects =          755                     Number of obs   =
755
No. of failures =          298
Time at risk    =        35110
                                                   LR chi2(13)     =
125.04
Log likelihood  =   -1723.9479                     Prob > chi2     =
0.0000

------------------------------------------------------------------------------
--------
          _t | Haz. Ratio   Std. Err.      z    P>|z|     [95% Conf.
Interval]
-------------
+----------------------------------------------------------------
         age |   1.036666   .0053741     6.95   0.000     1.026186
1.047252
      gender |   1.013791   .1250762     0.11   0.912      .796034
```

```
1.291116
          dm |   1.380852    .201975     2.21   0.027     1.036676
1.839293
         ckd |   1.795453   .2566332     4.09   0.000     1.356774
2.375968
          af |   1.532739   .1906768     3.43   0.001     1.201092
1.95596
         htn |   .9118639   .1493148    -0.56   0.573     .6615284
1.256931
       malig |    1.23673   .2459573     1.07   0.285     .8375104
1.826246
        copd |   1.090129   .1533033     0.61   0.539     .8275124
1.436088
          mi |   1.357652   .1936359     2.14   0.032     1.026563
1.795526
      stroke |   1.091736   .1610253     0.60   0.552     .8176548
1.457691
         pvd |    1.58427   .2359445     3.09   0.002     1.183205
2.121281
         cad |   .9681136   .1548408    -0.20   0.839     .7075946
1.324549
         hld |   .8412422   .1193487    -1.22   0.223     .6370292
1.11092
----------------------------------------------------------------------
--------

. *Model 4: Model 3 + medications

. stcox age gender dm ckd af htn malig copd mi stroke pvd cad hld dig
spiro hydra inotropes bb acearb if ra
> ce==2

         failure _d:  death == 1
   analysis time _t:  time

Iteration 0:   log likelihood = -1786.4702
Iteration 1:   log likelihood = -1723.9334
Iteration 2:   log likelihood = -1721.0024
Iteration 3:   log likelihood = -1720.9981
Iteration 4:   log likelihood = -1720.9981
Refining estimates:
Iteration 0:   log likelihood = -1720.9981

Cox regression -- Breslow method for ties

No. of subjects =          755                     Number of obs   =
755
No. of failures =          298
Time at risk    =        35110
                                                   LR chi2(19)     =
```

130.94

Log likelihood = -1720.9981 Prob > chi2 = 0.0000

| _t | Haz. Ratio | Std. Err. | z | P>\|z\| | [95% Conf. | Interval] |
|---|---|---|---|---|---|---|
| age | 1.035717 | .0055049 | 6.60 | 0.000 | 1.024984 | 1.046563 |
| gender | 1.016876 | .1273105 | 0.13 | 0.894 | .7956092 | 1.299681 |
| dm | 1.374835 | .2023596 | 2.16 | 0.031 | 1.0303 | 1.834584 |
| ckd | 1.693005 | .2537322 | 3.51 | 0.000 | 1.262083 | 2.27106 |
| af | 1.577653 | .2058923 | 3.49 | 0.000 | 1.221589 | 2.037502 |
| htn | .9311882 | .1544572 | -0.43 | 0.667 | .6727407 | 1.288924 |
| malig | 1.265775 | .2539731 | 1.17 | 0.240 | .8542131 | 1.875629 |
| copd | 1.094932 | .1561383 | 0.64 | 0.525 | .8279516 | 1.448002 |
| mi | 1.331421 | .1925178 | 1.98 | 0.048 | 1.002849 | 1.767647 |
| stroke | 1.077561 | .1603921 | 0.50 | 0.616 | .8049018 | 1.442584 |
| pvd | 1.553782 | .2325158 | 2.94 | 0.003 | 1.158809 | 2.083378 |
| cad | .9653408 | .1546176 | -0.22 | 0.826 | .7052525 | 1.321346 |
| hld | .8420266 | .1201688 | -1.20 | 0.228 | .6365721 | 1.113792 |
| dig | 1.012238 | .1509795 | 0.08 | 0.935 | .7556531 | 1.355948 |
| spiro | .8954301 | .1322463 | -0.75 | 0.455 | .6703746 | 1.19604 |
| hydra | 1.499277 | .2791935 | 2.17 | 0.030 | 1.040812 | 2.159691 |
| inotropes | .7104238 | .2127454 | -1.14 | 0.254 | .3950158 | 1.277675 |
| bb | .9968888 | .1795895 | -0.02 | 0.986 | .7003301 | 1.419027 |
| acearb | 1.003465 | .1654757 | 0.02 | 0.983 | .7263326 | 1.386338 |

```
. *Model 5: Model 4 + sodium

. stcox age gender dm ckd af htn malig copd mi stroke pvd cad hld dig
spiro hydra inotropes bb acearb na if
>  race==2

         failure _d:  death == 1
   analysis time _t:  time

Iteration 0:   log likelihood = -1786.4702
Iteration 1:   log likelihood = -1722.9231
Iteration 2:   log likelihood = -1719.9161
Iteration 3:   log likelihood = -1719.9111
Iteration 4:   log likelihood = -1719.9111
Refining estimates:
Iteration 0:   log likelihood = -1719.9111

Cox regression -- Breslow method for ties

No. of subjects =          755                     Number of obs   =
755
No. of failures =          298
Time at risk    =        35110
                                                   LR chi2(20)     =
133.12
Log likelihood  =   -1719.9111                     Prob > chi2     =
0.0000

----------------------------------------------------------------------
--------
          _t | Haz. Ratio   Std. Err.      z    P>|z|     [95% Conf.
Interval]
-------------
+----------------------------------------------------------------
         age |   1.035695   .0055083     6.59   0.000     1.024955
1.046547
      gender |   1.015851    .127349     0.13   0.900     .7945506
1.298787
          dm |    1.37966   .2033953     2.18   0.029     1.033438
1.841874
         ckd |   1.679721   .2515411     3.46   0.001     1.252472
2.252714
          af |   1.598746   .2095325     3.58   0.000     1.236575
2.06699
         htn |   .9505534   .1580646    -0.30   0.760     .6861718
1.316801
       malig |   1.283263   .2579992     1.24   0.215     .8653304
1.903045
        copd |   1.103678   .1575525     0.69   0.490     .8343174
```

```
1.460001
          mi |   1.333248   .1928863     1.99   0.047     1.004071
1.770344
      stroke |   1.048461   .1581402     0.31   0.754     .7801267
1.409094
         pvd |   1.527112   .2302966     2.81   0.005     1.136332
2.05228
         cad |    .961384   .1539633    -0.25   0.806     .7023912
1.315875
         hld |   .8296957   .1190704    -1.30   0.193     .6262707
1.099197
         dig |   1.005835   .1500512     0.04   0.969     .7508335
1.34744
       spiro |      .9104   .1350288    -0.63   0.527     .6807437
1.217533
       hydra |   1.534795   .2864272     2.30   0.022     1.064626
2.212604
   inotropes |   .7100618   .2125018    -1.14   0.253     .3949619
1.276548
          bb |   1.024886   .1859881     0.14   0.892     .7181355
1.462665
      acearb |   .9942878    .164171    -0.03   0.972     .7193938
1.374224
          na |    .966795   .0203493    -1.60   0.109     .9277225
1.007513
----------------------------------------------------------------------
--------

. *Model 6: Model 5 + creatinine

. stcox age gender dm ckd af htn malig copd mi stroke pvd cad hld dig
spiro hydra inotropes bb acearb na cr
>  if race==2

         failure _d:  death == 1
   analysis time _t:  time

Iteration 0:   log likelihood = -1784.9214
Iteration 1:   log likelihood = -1714.7558
Iteration 2:   log likelihood = -1710.4206
Iteration 3:   log likelihood = -1710.4117
Iteration 4:   log likelihood = -1710.4117
Refining estimates:
Iteration 0:   log likelihood = -1710.4117

Cox regression -- Breslow method for ties

No. of subjects =          753                     Number of obs   =
753
No. of failures =          298
```

Time at risk = 34925

LR chi2(21) = 149.02

Log likelihood = -1710.4117

Prob > chi2 = 0.0000

| _t | Haz. Ratio | Std. Err. | z | P>\|z\| | [95% Conf. | Interval] |
|---|---|---|---|---|---|---|
| age | 1.039846 | .0057643 | 7.05 | 0.000 | 1.028609 | 1.051205 |
| gender | .972112 | .1230301 | -0.22 | 0.823 | .7585576 | 1.245788 |
| dm | 1.263633 | .1886642 | 1.57 | 0.117 | .943048 | 1.693198 |
| ckd | 1.274813 | .2131705 | 1.45 | 0.147 | .9185679 | 1.769221 |
| af | 1.648905 | .2173297 | 3.79 | 0.000 | 1.273518 | 2.134942 |
| htn | .9843308 | .1625511 | -0.10 | 0.924 | .7121553 | 1.360528 |
| malig | 1.276701 | .2570611 | 1.21 | 0.225 | .8604022 | 1.894423 |
| copd | 1.088504 | .1566508 | 0.59 | 0.556 | .8209756 | 1.443211 |
| mi | 1.302003 | .1884717 | 1.82 | 0.068 | .9803843 | 1.729131 |
| stroke | .9869078 | .1505447 | -0.09 | 0.931 | .7318679 | 1.330823 |
| pvd | 1.566217 | .2359203 | 2.98 | 0.003 | 1.165829 | 2.104112 |
| cad | .9753015 | .1559493 | -0.16 | 0.876 | .7129072 | 1.334273 |
| hld | .8640514 | .1247056 | -1.01 | 0.311 | .6511612 | 1.146544 |
| dig | 1.08876 | .1649034 | 0.56 | 0.574 | .8091136 | 1.465059 |
| spiro | .9884605 | .1491457 | -0.08 | 0.939 | .7354009 | 1.328601 |
| hydra | 1.509118 | .2803535 | 2.22 | 0.027 | 1.048559 | 2.171969 |
| inotropes | .73943 | .2220358 | -1.01 | 0.315 | .4104862 | 1.331974 |
| bb | .9939386 | .1805239 | -0.03 | 0.973 | .696242 | 1.418923 |
| acearb | 1.05843 | .175202 | 0.34 | 0.732 | .7651788 | 1.464069 |

```
          na |   .9680334   .0205851    -1.53   0.127     .9285166
1.009232
          cr |   1.165714   .0403873     4.43   0.000     1.089184
1.247621
----------------------------------------------------------------------
--------

. *Model 7: Model 6 + ejection fraction

. stcox age gender dm ckd af htn malig copd mi stroke pvd cad hld dig
spiro hydra inotropes bb acearb na cr
>  ef if race==2

         failure _d:  death == 1
   analysis time _t:  time

Iteration 0:   log likelihood = -1630.4377
Iteration 1:   log likelihood = -1564.2205
Iteration 2:   log likelihood = -1561.3736
Iteration 3:   log likelihood = -1561.3694
Iteration 4:   log likelihood = -1561.3694
Refining estimates:
Iteration 0:   log likelihood = -1561.3694

Cox regression -- Breslow method for ties

No. of subjects =          704                     Number of obs   =
704
No. of failures =          276
Time at risk    =        32234
                                                   LR chi2(22)     =
138.14
Log likelihood  =   -1561.3694                     Prob > chi2     =
0.0000

----------------------------------------------------------------------
--------
          _t | Haz. Ratio   Std. Err.      z    P>|z|     [95% Conf.
Interval]
-------------
+----------------------------------------------------------------
         age |   1.038298   .0059554     6.55   0.000     1.026691
1.050036
      gender |   .9328986   .1254036    -0.52   0.605     .7168245
1.214104
          dm |   1.151525   .1801982     0.90   0.367     .8473672
1.564858
         ckd |   1.273777   .2188264     1.41   0.159     .9096254
1.783709
          af |   1.844755   .2569926     4.40   0.000      1.40397
```

```
2.423927
         htn |   1.055869    .183821     0.31   0.755     .7506204
1.48525
       malig |   1.275205   .2657887     1.17   0.243     .8475501
1.918644
        copd |   1.164601   .1735472     1.02   0.307     .8696257
1.559632
          mi |   1.231243   .1859556     1.38   0.168     .9157694
1.655394
      stroke |    .962785   .1528467    -0.24   0.811     .7053376
1.3142
         pvd |    1.68526   .2665827     3.30   0.001     1.236004
2.297811
         cad |   1.004241   .1675817     0.03   0.980     .7240937
1.392777
         hld |   .8301725   .1239833    -1.25   0.213     .6195044
1.11248
         dig |   1.025592   .1645332     0.16   0.875     .7488907
1.404529
       spiro |   .9911021   .1598459    -0.06   0.956     .7224965
1.359568
       hydra |   1.470551   .2804941     2.02   0.043     1.011861
2.137169
   inotropes |   .7364517   .2262225    -1.00   0.319     .4033416
1.344669
          bb |   .9386098   .1798164    -0.33   0.741     .6447838
1.366331
      acearb |   .9615094   .1713311    -0.22   0.826     .6780757
1.363418
          na |   .9677712   .0213463    -1.49   0.137     .9268246
1.010527
          cr |    1.16492   .0414151     4.29   0.000     1.086511
1.248987
          ef |   .9958228   .0047935    -0.87   0.385      .986472
1.005262
----------------------------------------------------------------------
--------

.
. *Other*

. *Model 2: Race + demographics (age, gender)

. stcox age gender if race==3

         failure _d:  death == 1
   analysis time _t:  time

Iteration 0:   log likelihood = -3579.7299
Iteration 1:   log likelihood = -3550.3985
```

```
Iteration 2:   log likelihood = -3550.2432
Iteration 3:   log likelihood = -3550.2431
Refining estimates:
Iteration 0:   log likelihood = -3550.2431

Cox regression -- Breslow method for ties

No. of subjects =          813                     Number of obs   =
813
No. of failures =          588
Time at risk    =        28147
                                                   LR chi2(2)      =
58.97
Log likelihood  =   -3550.2431                     Prob > chi2     =
0.0000

----------------------------------------------------------------------
--------
          _t | Haz. Ratio   Std. Err.      z    P>|z|     [95% Conf.
Interval]
-------------
+----------------------------------------------------------------
         age |   1.021379    .003164     6.83   0.000     1.015196
1.027599
      gender |    .895099   .0767543    -1.29   0.196     .7566254
1.058915
----------------------------------------------------------------------
--------

. *Model 3: Model 2 + comorbidities

. stcox age gender dm ckd af htn malig copd mi stroke pvd cad hld if
race==3

         failure _d:  death == 1
   analysis time _t:  time

Iteration 0:   log likelihood = -3579.7299
Iteration 1:   log likelihood = -3530.2071
Iteration 2:   log likelihood = -3529.0336
Iteration 3:   log likelihood = -3529.0328
Refining estimates:
Iteration 0:   log likelihood = -3529.0328

Cox regression -- Breslow method for ties

No. of subjects =          813                     Number of obs   =
813
No. of failures =          588
Time at risk    =        28147
```

```
                                                  LR chi2(13)     =
101.39
Log likelihood  =   -3529.0328                    Prob > chi2     =
0.0000

----------------------------------------------------------------------
--------
          _t | Haz. Ratio   Std. Err.      z    P>|z|     [95% Conf.
Interval]
-------------
+----------------------------------------------------------------
         age |   1.018774   .0034123     5.55   0.000     1.012108
1.025484
      gender |   .9028705   .0799081    -1.15   0.248     .7590849
1.073892
          dm |   1.214689   .1142173     2.07   0.039     1.010245
1.460507
         ckd |    1.39593   .1663659     2.80   0.005     1.105142
1.763231
          af |   1.136543   .1036962     1.40   0.161     .9504376
1.359089
         htn |   1.059753   .1087631     0.57   0.572     .8666529
1.295877
       malig |   1.525925   .2232333     2.89   0.004     1.145533
2.032631
        copd |    1.34044   .1233114     3.18   0.001      1.11929
1.605285
          mi |   1.134568   .1158503     1.24   0.216     .9287839
1.385946
      stroke |   1.048989    .126661     0.40   0.692     .8279272
1.329077
         pvd |   1.065647   .1273515     0.53   0.595     .8431202
1.346906
         cad |   .9943224   .1051242    -0.05   0.957     .8082289
1.223264
         hld |   1.099971    .132493     0.79   0.429     .8686665
1.392867
----------------------------------------------------------------------
--------

. *Model 4: Model 3 + medications

. stcox age gender dm ckd af htn malig copd mi stroke pvd cad hld dig
spiro hydra inotropes bb acearb if ra
> ce==3

         failure _d:  death == 1
   analysis time _t:  time

Iteration 0:   log likelihood = -3579.7299
```

```
Iteration 1:   log likelihood = -3527.2659
Iteration 2:   log likelihood = -3525.9113
Iteration 3:   log likelihood = -3525.9102
Refining estimates:
Iteration 0:   log likelihood = -3525.9102

Cox regression -- Breslow method for ties

No. of subjects =          813                     Number of obs   =
813
No. of failures =          588
Time at risk    =        28147
                                                   LR chi2(19)     =
107.64
Log likelihood  =   -3525.9102                     Prob > chi2     =
0.0000

----------------------------------------------------------------------
--------
          _t | Haz. Ratio   Std. Err.      z    P>|z|     [95% Conf.
Interval]
-------------
+-----------------------------------------------------------------
         age |   1.019377   .0035076     5.58   0.000     1.012526
1.026275
      gender |   .8828356   .0791792    -1.39   0.165     .7405218
1.052499
          dm |   1.243709   .1180504     2.30   0.022     1.032582
1.498005
         ckd |    1.46954    .180978     3.13   0.002     1.154393
1.870722
          af |   1.080369   .1062535     0.79   0.432     .8909576
1.310048
         htn |   1.052797   .1094424     0.49   0.621      .858735
1.290716
       malig |   1.583758   .2335512     3.12   0.002      1.18622
2.114523
        copd |   1.315398   .1241034     2.91   0.004     1.093324
1.582578
          mi |   1.139771   .1175915     1.27   0.205     .9311036
1.395202
      stroke |    1.05049   .1274697     0.41   0.685     .8281413
1.332537
         pvd |   1.063115   .1278945     0.51   0.611     .8398065
1.345802
         cad |   1.014801   .1082876     0.14   0.890     .8232858
1.250867
         hld |   1.081996   .1308359     0.65   0.515     .8536847
1.371367
         dig |   1.127711   .1115796     1.21   0.224     .9289175
```

```
1.369048
       spiro |    1.20456   .1228352     1.83   0.068     .9863403
1.471058
       hydra |   .9847993    .146937    -0.10   0.918     .7350962
1.319323
   inotropes |   .8746469   .1274169    -0.92   0.358     .6574024
1.163682
          bb |   .9709064   .1108578    -0.26   0.796     .7762245
1.214416
      acearb |   .9864348   .0999378    -0.13   0.893     .8087817
1.20311
----------------------------------------------------------------------
--------

. *Model 5: Model 4 + sodium

. stcox age gender dm ckd af htn malig copd mi stroke pvd cad hld dig
spiro hydra inotropes bb acearb na if
>  race==3

         failure _d:  death == 1
   analysis time _t:  time

Iteration 0:   log likelihood = -3579.7299
Iteration 1:   log likelihood = -3527.1849
Iteration 2:   log likelihood = -3525.8265
Iteration 3:   log likelihood = -3525.8253
Refining estimates:
Iteration 0:   log likelihood = -3525.8253

Cox regression -- Breslow method for ties

No. of subjects =          813                     Number of obs   =
813
No. of failures =          588
Time at risk    =        28147
                                                   LR chi2(20)     =
107.81
Log likelihood  =   -3525.8253                     Prob > chi2     =
0.0000

----------------------------------------------------------------------
--------
          _t | Haz. Ratio   Std. Err.      z    P>|z|     [95% Conf.
Interval]
-------------
+-----------------------------------------------------------------
         age |   1.019238   .0035222     5.51   0.000     1.012358
1.026165
      gender |    .881464   .0791418    -1.41   0.160     .7392305
```

```
1.051064
          dm |   1.244401   .1181322     2.30   0.021     1.033129
1.498876
         ckd |   1.462111   .1809265     3.07   0.002     1.147229
1.863421
          af |    1.07951   .1061331     0.78   0.436     .8903074
1.308921
         htn |   1.053494   .1095501     0.50   0.616     .8592466
1.291654
       malig |   1.589965   .2349885     3.14   0.002     1.190103
2.124177
        copd |   1.315885   .1241746     2.91   0.004     1.093688
1.583223
          mi |   1.139095   .1175443     1.26   0.207     .9305157
1.394429
      stroke |   1.053819   .1281744     0.43   0.666     .8303009
1.337508
         pvd |   1.058693   .1278214     0.47   0.637     .8356034
1.341343
         cad |   1.018332   .1089499     0.17   0.865     .8256964
1.255909
         hld |   1.084687   .1313246     0.67   0.502     .8555561
1.375184
         dig |   1.128202   .1116679     1.22   0.223      .929258
1.369739
       spiro |   1.202864   .1227394     1.81   0.070     .9848281
1.469171
       hydra |   .9871038   .1473455    -0.09   0.931     .7367218
1.322581
   inotropes |   .8711528   .1272741    -0.94   0.345     .6542369
1.159988
          bb |   .9702603   .1108132    -0.26   0.792     .7756624
1.213679
      acearb |   .9838682   .0998822    -0.16   0.873     .8063489
1.200469
          na |   .9917165   .0199166    -0.41   0.679      .953439
1.031531
----------------------------------------------------------------------
--------

. *Model 6: Model 5 + creatinine

. stcox age gender dm ckd af htn malig copd mi stroke pvd cad hld dig
spiro hydra inotropes bb acearb na cr
>  if race==3

         failure _d:  death == 1
   analysis time _t:  time

Iteration 0:   log likelihood = -3578.1741
```

```
Iteration 1:   log likelihood = -3513.5731
Iteration 2:   log likelihood = -3511.7765
Iteration 3:   log likelihood = -3511.7748
Refining estimates:
Iteration 0:   log likelihood = -3511.7748

Cox regression -- Breslow method for ties

No. of subjects =          812                     Number of obs   =
812
No. of failures =          588
Time at risk    =        28050
                                                   LR chi2(21)     =
132.80
Log likelihood  =   -3511.7748                     Prob > chi2     =
0.0000

----------------------------------------------------------------------
--------
          _t | Haz. Ratio   Std. Err.      z    P>|z|     [95% Conf.
Interval]
-------------
+-----------------------------------------------------------------
         age |   1.021424   .0035878     6.03   0.000     1.014416
1.02848
      gender |   .8540813   .0767808    -1.75   0.079      .716106
1.018641
          dm |   1.209568   .1145346     2.01   0.045     1.004684
1.456234
         ckd |   1.192663    .154767     1.36   0.175     .9248272
1.538065
          af |   1.101188   .1085583     0.98   0.328     .9077105
1.335904
         htn |   1.124121   .1170174     1.12   0.261     .9166549
1.378544
       malig |   1.647266   .2438239     3.37   0.001     1.232455
2.20169
        copd |   1.406637   .1344048     3.57   0.000     1.166405
1.696347
          mi |   1.117849   .1157551     1.08   0.282     .9125149
1.369388
      stroke |   1.042373    .126454     0.34   0.732       .82179
1.322164
         pvd |   .9722712   .1183004    -0.23   0.817     .7659812
1.234118
         cad |   .9804558    .104982    -0.18   0.854     .7948513
1.2094
         hld |   1.074162   .1304866     0.59   0.556     .8465799
1.362924
         dig |    1.17761   .1178109     1.63   0.102     .9679332
```

```
1.432708
       spiro |   1.291052   .1339491     2.46   0.014     1.053489
1.582186
       hydra |   .9274194   .1378876    -0.51   0.612     .6929798
1.241171
   inotropes |   .9363391   .1376828    -0.45   0.655     .7018903
1.2491
          bb |   .9353206   .1068196    -0.59   0.558     .7477353
1.169966
      acearb |   1.038052   .1061745     0.37   0.715     .8494856
1.268475
          na |   .9953469   .0198613    -0.23   0.815      .957171
1.035046
          cr |   1.158419   .0310113     5.49   0.000     1.099205
1.220823
----------------------------------------------------------------------
--------

. *Model 7: Model 6 + ejection fraction

. stcox age gender dm ckd af htn malig copd mi stroke pvd cad hld dig
spiro hydra inotropes bb acearb na cr
>  ef if race==3

         failure _d:  death == 1
   analysis time _t:  time

Iteration 0:   log likelihood = -2502.5799
Iteration 1:   log likelihood = -2451.0673
Iteration 2:   log likelihood = -2449.6756
Iteration 3:   log likelihood = -2449.6747
Refining estimates:
Iteration 0:   log likelihood = -2449.6747

Cox regression -- Breslow method for ties

No. of subjects =          600                     Number of obs   =
600
No. of failures =          435
Time at risk    =        20198
                                                   LR chi2(22)     =
105.81
Log likelihood  =   -2449.6747                     Prob > chi2     =
0.0000

----------------------------------------------------------------------
--------
          _t | Haz. Ratio   Std. Err.      z    P>|z|     [95% Conf.
Interval]
-------------
```

```
+----------------------------------------------------------------
         age |    1.02325   .0043403     5.42   0.000     1.014778
1.031792
      gender |   .8042872   .0841736    -2.08   0.037     .
6551304    .9874033
          dm |   1.202781   .1360242     1.63   0.103     .9636581
1.50124
         ckd |   1.364023   .1930606     2.19   0.028     1.033581
1.800109
          af |   1.016837   .1186573     0.14   0.886     .8089511
1.278145
         htn |   1.208625    .152831     1.50   0.134     .9433156
1.548554
       malig |    1.50409   .2655598     2.31   0.021     1.064111
2.125987
        copd |   1.426909    .162997     3.11   0.002     1.140678
1.784965
          mi |   1.085465   .1285669     0.69   0.489     .8605899
1.369102
      stroke |   1.008767   .1377723     0.06   0.949     .7718594
1.31839
         pvd |   1.037293   .1371069     0.28   0.782     .8005557
1.344037
         cad |   .9270804   .1197513    -0.59   0.558     .7197264
1.194173
         hld |   1.008266   .1338458     0.06   0.951     .7772837
1.307889
         dig |   1.206958   .1426745     1.59   0.112     .9573517
1.521642
       spiro |   1.390961   .1716909     2.67   0.008     1.092064
1.771664
       hydra |   .8932879   .1428136    -0.71   0.480     .6529897
1.222015
   inotropes |   .8302587   .1461314    -1.06   0.291     .5880252
1.172279
          bb |   .9310932   .1373323    -0.48   0.628       .69734
1.243202
      acearb |   1.145769   .1405224     1.11   0.267     .9009519
1.45711
          na |   .9754735   .0247689    -0.98   0.328     .9281155
1.025248
          cr |   1.149066   .0365472     4.37   0.000     1.079622
1.222977
          ef |   .9990526   .0034074    -0.28   0.781     .9923965
1.005753
----------------------------------------------------------------------
--------

.
. *A couple more models by EF (HFrEF vs HFpEF)*
```

```
. gen hfgroup = .
(11957 missing values generated)

. replace hfgroup = 0 if ef<40 & ef!=.
(3885 real changes made)

. replace hfgroup = 1 if ef>=40 & ef!=.
(5896 real changes made)

.
. stcox i.race age gender dm ckd af htn malig copd mi stroke pvd cad
hld dig spiro hydra inotropes bb acear
> b na cr if hfgroup==0

         failure _d:  death == 1
   analysis time _t:  time

Iteration 0:   log likelihood = -4839.5042
Iteration 1:   log likelihood = -4688.6126
Iteration 2:   log likelihood = -4682.7989
Iteration 3:   log likelihood =  -4682.788
Iteration 4:   log likelihood =  -4682.788
Refining estimates:
Iteration 0:   log likelihood =  -4682.788

Cox regression -- Breslow method for ties

No. of subjects =         1325                     Number of obs   =
1325
No. of failures =          733
Time at risk    =        50018
                                                   LR chi2(24)     =
313.43
Log likelihood  =    -4682.788                     Prob > chi2     =
0.0000

----------------------------------------------------------------------
--------
          _t | Haz. Ratio   Std. Err.      z    P>|z|     [95% Conf.
Interval]
-------------
+-----------------------------------------------------------------
        race |
  Caucasian  |   .9787355   .1001766    -0.21   0.834     .8008335
1.196158
   Hispanic  |   .5263622   .0634553    -5.32   0.000     .
4155934    .6666544
      Other  |   1.289351   .1324852     2.47   0.013     1.054162
1.577012
```

```
             |
         age |   1.030497   .0032835     9.43   0.000     1.024082
1.036953
      gender |   1.069789   .0860752     0.84   0.402     .9137147
1.252523
          dm |   1.134791   .0912379     1.57   0.116     .9693455
1.328474
         ckd |   1.361786   .1437545     2.93   0.003      1.10727
1.674806
          af |    1.20099    .098363     2.24   0.025      1.02288
1.410114
         htn |   .9066221   .0818116    -1.09   0.277     .7596538
1.082024
       malig |   1.269226    .157426     1.92   0.055     .9953178
1.618513
        copd |   1.211299   .1059057     2.19   0.028     1.020539
1.437717
          mi |   1.080882   .0948686     0.89   0.376     .9100574
1.283772
      stroke |   .9133697   .1047373    -0.79   0.429     .7295217
1.143549
         pvd |   1.413524   .1355729     3.61   0.000     1.171287
1.705858
         cad |   1.122134   .1140416     1.13   0.257     .9194707
1.369467
         hld |   .8909894   .0945425    -1.09   0.277     .7236888
1.096966
         dig |   1.070325   .0869399     0.84   0.403     .9127981
1.255038
       spiro |   .9813078    .083516    -0.22   0.825     .8305431
1.15944
       hydra |   1.032117   .1163441     0.28   0.779     .8275198
1.2873
   inotropes |    1.04836    .114117     0.43   0.664     .8469444
1.297676
          bb |   .9779181   .1254002    -0.17   0.862     .7605913
1.257343
      acearb |   1.031165   .1078812     0.29   0.769     .8399916
1.265848
          na |   .9833728   .0173078    -0.95   0.341     .9500286
1.017887
          cr |    1.14393    .029038     5.30   0.000     1.088409
1.202283
----------------------------------------------------------------------
--------

. stcox i.race age gender dm ckd af htn malig copd mi stroke pvd cad
hld dig spiro hydra inotropes bb acear
> b na cr if hfgroup==1
```

```
         failure _d:  death == 1
   analysis time _t:  time

Iteration 0:   log likelihood = -7837.8509
Iteration 1:   log likelihood = -7675.7832
Iteration 2:   log likelihood = -7672.2744
Iteration 3:   log likelihood = -7672.2719
Refining estimates:
Iteration 0:   log likelihood = -7672.2719

Cox regression -- Breslow method for ties

No. of subjects =         1812                   Number of obs   =
1812
No. of failures =         1145
Time at risk    =        68722
                                                 LR chi2(24)     =
331.16
Log likelihood  =   -7672.2719                   Prob > chi2     =
0.0000

----------------------------------------------------------------------
--------
         _t | Haz. Ratio   Std. Err.      z    P>|z|     [95% Conf.
Interval]
-------------
+------------------------------------------------------------------
       race |
  Caucasian  |   1.042079   .0863138     0.50   0.619     .8859253
1.225757
   Hispanic  |   .5715559   .0549014    -5.82   0.000     .
4734734    .6899566
      Other  |   1.538226   .1330633     4.98   0.000     1.298337
1.822438
            |
        age |   1.026732   .0026674    10.15   0.000     1.021517
1.031973
     gender |   .9786963   .0632748    -0.33   0.739     .8622157
1.110913
         dm |   1.097169   .0717764     1.42   0.156     .9651349
1.247265
        ckd |   1.306806   .1036463     3.37   0.001     1.118665
1.52659
         af |   1.108545    .077485     1.47   0.140     .9666206
1.271307
        htn |   .9641838   .0750205    -0.47   0.639     .8278092
1.123025
      malig |   1.337257   .1247776     3.11   0.002     1.113757
1.605607
       copd |   1.243921   .0821363     3.31   0.001     1.092919
```

```
1.415787
          mi |   1.173089   .0891383     2.10   0.036     1.010768
1.361477
      stroke |   1.256695   .1006243     2.85   0.004     1.074171
1.470232
         pvd |   1.150438   .0923511     1.75   0.081     .9829541
1.346459
         cad |   .8565318   .0650664    -2.04   0.041     .
7380436    .9940426
         hld |   .9191113   .0738753    -1.05   0.294     .7851474
1.075932
         dig |   1.162031   .0937364     1.86   0.063     .9920984
1.361071
       spiro |   1.275142   .1061919     2.92   0.004     1.083108
1.501224
       hydra |   1.058321   .1212838     0.49   0.621     .8454146
1.324846
   inotropes |   1.077489   .1632269     0.49   0.622     .8006926
1.449973
          bb |   .9388058   .0749701    -0.79   0.429     .8027892
1.097868
      acearb |   .9489428   .0643658    -0.77   0.440     .8308142
1.083868
          na |   .9857349   .0106662    -1.33   0.184     .9650497
1.006864
          cr |   1.089115   .0185199     5.02   0.000     1.053415
1.126025
----------------------------------------------------------------------
--------

.
. log close
      name:  <unnamed>
       log:  /Users/Renato/Desktop/Einstein/Einstein research/
Hyponatremia and HF/6.feb.log
  log type:  text
 closed on:   6 Feb 2018, 19:31:09
----------------------------------------------------------------------
-------------------------------------
```
